# Tuning the Catalytic Performance of Cobalt Nanoparticles by Tungsten Doping for Efficient and Selective Hydrogenation of Quinolines under Mild Conditions

**DOI:** 10.1021/acscatal.1c01561

**Published:** 2021-06-18

**Authors:** Marta Puche, Lichen Liu, Patricia Concepción, Iván Sorribes, Avelino Corma

**Affiliations:** Instituto de Tecnología Química, Universitat Politècnica de València-Consejo Superior de Investigaciones Científicas, Avenida de los Naranjos s/n, 46022 Valencia, Spain

**Keywords:** non-noble metal catalysts, CoW bimetallic alloys, heterogeneous catalysis, selective hydrogenation, quinolines

## Abstract

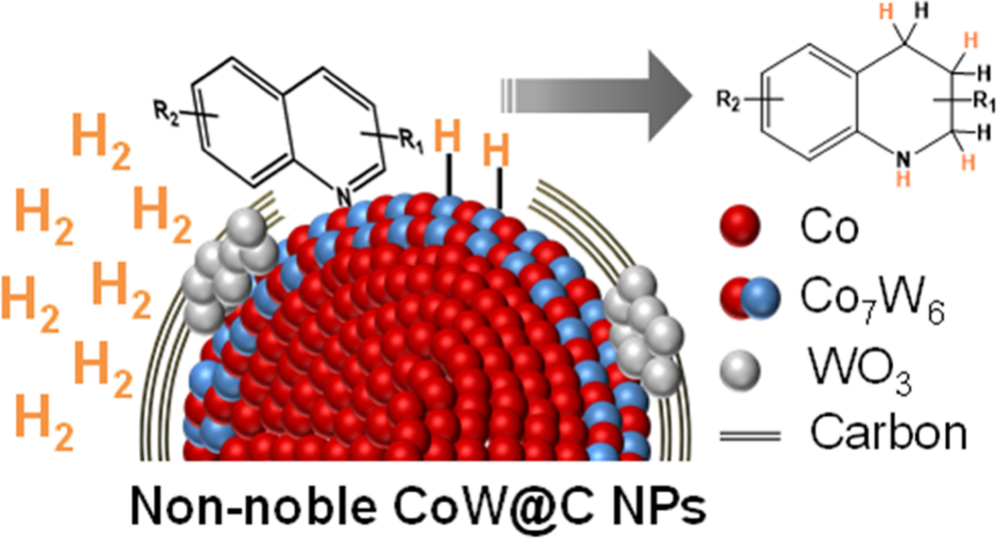

Non-noble bimetallic CoW nanoparticles (NPs) partially embedded in a carbon matrix (CoW@C) have been prepared by a facile hydrothermal carbon-coating methodology followed by pyrolysis under an inert atmosphere. The bimetallic NPs, constituted by a multishell core–shell structure with a metallic Co core, a W-enriched shell involving Co_7_W_6_ alloyed structures, and small WO_3_ patches partially covering the surface of these NPs, have been established as excellent catalysts for the selective hydrogenation of quinolines to their corresponding 1,2,3,4-tetrahydroquinolines under mild conditions of pressure and temperature. It has been found that this bimetallic catalyst displays superior catalytic performance toward the formation of the target products than the monometallic Co@C, which can be attributed to the presence of the CoW alloyed structures.

## Introduction

Catalytic hydrogenation by non-noble metal nanoparticles (NPs) has attracted increasing interest in the research laboratories and for industrial applications.^1−4^ Indeed, from the perspective of sustainability, it has become an essential methodology for reductive transformations of chemical substances.^5^ Since activity and selectivity of metal NPs can be tuned by controlling their size, shape, composition, and support interactions, hydrogenation reactions can be performed avoiding or minimizing the formation of byproducts.^6−21^ Fundamentally, this is particularly advantageous for the preparation of 1,2,3,4-tetrahydroquinolines, which are important building blocks broadly present in many bioactive compounds including natural products, agrochemicals, synthetic drugs, and lead compounds.^22−24^ These industrially valuable scaffolds can be conveniently synthesized by partial hydrogenation of readily available quinoline derivatives, a synthetic methodology with high atom-economy if the formation of completely saturated products, as well as hydrogenation of other co-existing reducible functionalities, are avoided.^25,26^ In addition to selectivity issues, this hydrogenative transformation also involves other scientific and technological challenges, such as high reaction energy barriers to break aromaticity and prevent catalyst poisoning.^27,28^

Impressive achievements have been reached in this area by applying catalysts based on noble metal NPs (e.g., Pd,^29−37^ Pt,^38−41^ Rh,^38,42−50^ Ru,^41,51−64^ Ir,^38,65,66^ and Au^36,67^). However, the high and volatile price associated with the low availability of precious metals has boosted the interest to exploit cheap and earth-abundant metals for the development of novel catalysts in recent times, although, to date, to a limited extent for the hydrogenation of quinolines. The earliest work dealt with the use of the traditional Raney-Ni catalyst.^1,68−70^ In 2015, Beller and co-workers prepared a cobalt-based catalyst consisting of N-graphene-modified cobalt oxide/cobalt NPs by pyrolysis of a nonvolatile phenanthroline-ligated complex on alumina (Co_3_O_4_–Co/NGr@α-Al_2_O_3_), and successfully applied for the hydrogenation of N-heteroarenes including quinoline compounds.^71^ Later, the groups of Wang^72^ and Li^73^ developed other related N-doped graphene-coated cobalt (oxide) NPs avoiding the use of sophisticated ligands and investigated their catalytic performance for the title reaction.

In 2018, we hydrothermally prepared cobalt–molybdenum–sulfide (Co–Mo–S) catalysts with tunable phase composition that display an optimal and broad-spectrum performance for the chemo- and regioselective hydrogenation of quinoline derivatives.^74^ Shortly after, it was demonstrated that a wide range of functionalized 1,2,3,4-tetrahydroquinolines can also be accessed by applying N-doped carbon-modified iron-based catalysts, which resulted from pyrolysis of a carbon-impregnated composite obtained from an iron salt and N-aryliminopyridines as ligands.^75^ More recently, MOF-derived N-coordinated cobalt nanocrystals,^76^ Co–N*_x_* sites previously liberated from N-doped carbon nanotubes (N-CNTs) under laser irradiation by the liquid technique,^77^ in situ generated Co NPs by hydrolysis of NaBH_4_,^78^ copper oxide NPs supported on alumina,^79^ and bimetallic CoCu oxide NPs,^80^ have also been applied for the hydrogenation of various quinolines. Meanwhile, this hydrogenative transformation catalyzed by non-precious metal NPs-based catalysts (Cu,^81^ Ni,^82^ and Co^83,84^) has also been investigated from the perspective of H_2_-storage systems based on liquid organic hydrogen carriers.

It is also noteworthy that other reductive catalytic protocols catalyzed by non-noble metal-based heterogeneous catalysts, including transfer hydrogenation (with formic acid^85−87^ or ammonia borane^88−90^), photocatalytic hydrogenation,^91^ and sequential dearomative hydroboration/hydrogenation^92^ have been proposed for the synthesis of 1,2,3,4-tetrahydroquinolines. However, the practical simplicity and higher atom efficiency make the straightforward hydrogenation with molecular hydrogen more advantageous.

In spite of the great success achieved for the hydrogenation of quinoline derivatives catalyzed by non-noble metals, more efficient catalysts are required for practical applications. A constant target for substituting precious by non-noble metals has been to achieve high activities similar to those of the formers under mild reaction conditions while maintaining good stability. In contrast, the non-noble NPs-based catalysts reported to date for the hydrogenation of quinolines require demanding conditions of H_2_ pressure, temperature, and/or long reaction times; therefore, more advanced high-performance catalysts are required.

The hydrogenation of N-heteroarene compounds, including quinoline derivatives, is a key reaction step that takes place before C–N bond breaking in hydro-denitrogenation (HDN) treatments, routinely applied in the refining industry for removing nitrogen heteroatoms from crude feedstocks. Bimetallic sulfides, constituted by a combination of group VI metals (either Mo or W) with group VIII metals (such as Co and Ni), supported on acidic substrates are the most commonly used catalysts in the industry for hydrotreatment processes.^93^ It is generally accepted that in these systems Co or Ni atoms decorate the edge position of MoS_2_ or WS_2_ forming Co(Ni)–W(Mo)–S structures that display “brim” sites involving metal-like electronic states where N-containing compounds tend to be adsorbed and activated.^94−96^ Interestingly, the promoting role of Co and Ni has also been observed in sulfur-free Mo- and W-based oxide catalysts applied for hydroconversion reactions of hydrocarbons, which have the advantage of avoiding deactivation by desulfurization under hydrogenative conditions. With high controversy, there is a strong tendency to attribute this synergy effect to the formation of β-Co(Ni)W(Mo)O_4_ species that promote a better electronic interaction between the metals (W, Mo) and the promoters (Co, Ni), thus favoring the reductive and acid properties of the mixed oxide catalysts.^97−100^

Inspired by industry, in this work, we have developed a series of Co- and W-based bimetallic materials (CoW@C) and have applied them for the selective hydrogenation of quinoline (**1a**) to afford the partially hydrogenated product 1,2,3,4-tetrahydroquinoline (**2a**). We have seen that the activity of Co NPs is enhanced by the presence of surface CoW alloyed species, while the presence of metal oxides decreases their catalytic performance. Notably, under otherwise the same reaction conditions, the most active catalyst CoW@C-0.05 displays higher catalytic activity (determined by comparison of the initial reaction rates normalized to the mass of metal weights) than previously reported Co-based catalysts. Comparative H_2_–D_2_ exchange experiments suggest that a homolytic rather than a heterolytic dissociation of H_2_ takes place on the metallic surface, which is proposed to be the active site where **1a** is also adsorbed. The surface reaction between both the activated reactants is the rate-determining step in the hydrogenation process, as revealed by kinetic experiments performed with the catalyst CoW@C-0.05. Furthermore, we have demonstrated that the use of this catalyst allows the preparation of a broad range of 1,2,3,4-tetrahydroquinolines under mild conditions of both temperature and pressure, thus constituting a real alternative to precious metal-based catalysts for practical applications.

## Results and Discussion

### Synthesis and Characterization of CoW@C Materials

In recent years, our group has been engaged in research devoted to the preparation of Co NPs and their application as catalysts for selective hydrogenation of fine chemicals for substituting noble metals with non-noble ones. In 2016, we reported monodispersed Co NPs coated with carbon layers, prepared by thermal decomposition of a Co–EDTA complex, that displayed higher catalytic activity than previously reported heterogeneous non-noble metal and the state-of-the-art Au catalysts for the hydrogenation of substituted nitroarenes into the corresponding anilines under mild conditions.^101^ Next, we prepared improved catalysts based on Co NPs by a simple carbon coating process consisting of a hydrothermal treatment with glucose as a carbon source, followed by pyrolysis.^102,103^ The resultant NPs stabilized by a few layers of carbons (Co@C) show enhanced catalytic activity for the hydrogenation of nitroarenes to the corresponding anilines and for the hydrogenation of levulinic acid to γ-valerolactone. Particularly worth mentioning is the fact that the carbon-coating method used for the preparation of these non-noble metal-based nanoparticulate materials also enables the synthesis of bimetallic NPs. Indeed, CoNi@C NPs were prepared with significantly higher catalytic activity than the monometallic ones (Co@C NPs), while maintaining high selectivity for the chemoselective hydrogenation of nitroarenes.^102^

**Scheme 1:**
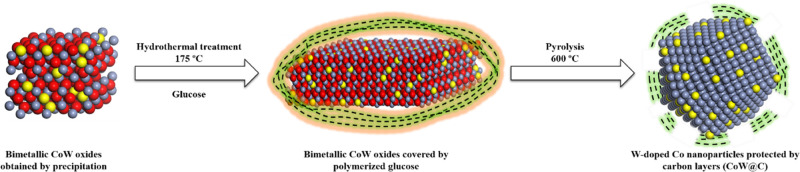
Schematic Representation of the Synthesis of Bimetallic CoW NPs Partially Coated by Carbon Layers (CoW@C) Different types of atoms are indicated by different colors: cobalt (gray), tungsten (yellow), and oxygen (red).

In this work, we developed a hydrothermal carbon-coating methodology for the preparation of bimetallic CoW@C materials (Scheme 1). First, bimetallic oxides were obtained by slowly dripping an aqueous solution of sodium carbonate into an ethylene glycol mixture of cobalt(II) acetate and different amounts of sodium tungstate at 165 °C. The resultant solids were coated by glucose through a hydrothermal treatment in an autoclave at 175 °C and subsequently pyrolyzed under an inert atmosphere at 600 °C (see the Experimental Section for the extended preparation details). At such a high temperature, according to the so-called carbochemical reduction,^104^ the bimetallic oxides are expected to be reduced by carbon from glucose, while some of the carbon is oxidized to CO_2_. Importantly, the rest of the carbon will tend to be graphitized into thin layers, resulting in the partial covering of the nanoparticles. According to our previous studies, the role of the carbon layers is to protect the non-noble metal NPs from agglomeration and from deep oxidation by air, as well as to promote the in situ reduction of oxide species on the surface of the NPs under reaction conditions.^101−103,105^ The resultant bimetallic catalysts were denoted CoW@C-*X*, where *X* indicates the W/Co mole ratio used in the catalyst preparation. For comparison, monometallic Co@C NPs were also synthesized following the same preparation methodology.

**Figure 1:**
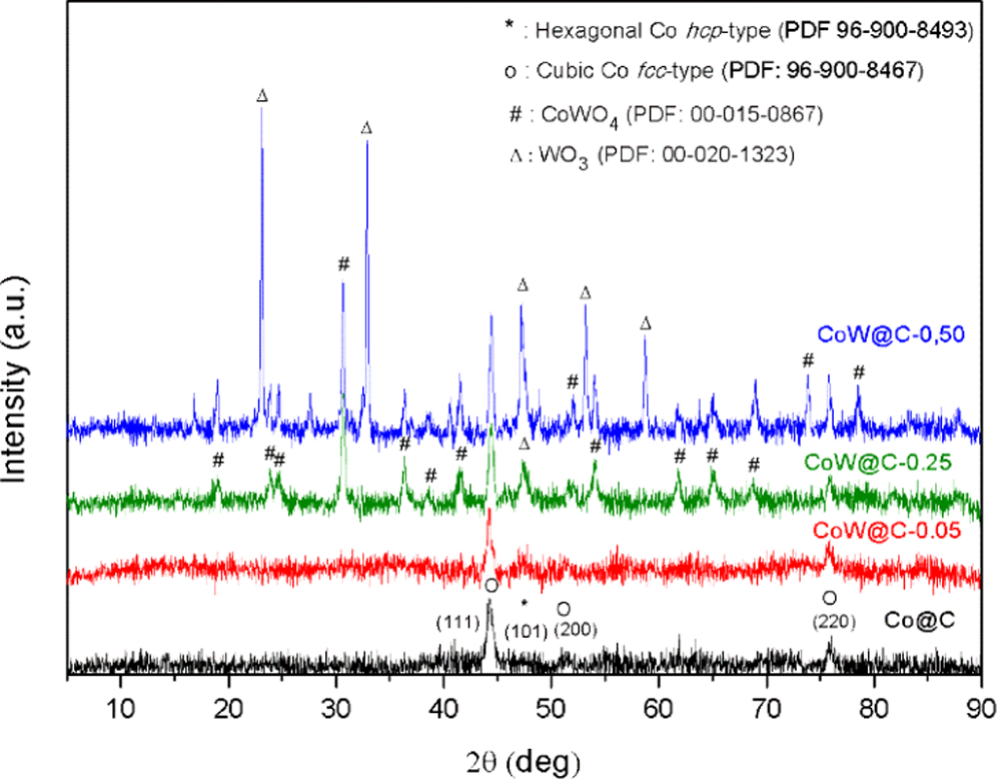
XRD diffraction patterns of the monometallic Co@C and bimetallic CoW@C materials.

X-ray diffraction (XRD) patterns of the prepared bimetallic CoW@C materials are shown in Figure 1. The XRD pattern of the material with the lowest content of tungsten, named CoW@C-0.05, is dominated by the presence of diffraction peaks at 2θ values of 44.2, 51.5, and 75.8° corresponding to the (111), (200) and (220) planes, respectively, of the cubic Co (face-centered cubic (fcc) type, PDF code: 96-900-8467) phase. In addition, other weak diffraction peaks at 41.6 and 47.5°, characteristic of the (100) and (101) planes, respectively, of the hexagonal Co (hexagonal close-packed (hcp) type, PDF code 96-900-8493) phase, are also slightly visible. As a result of the low amount of tungsten doping, no additional peaks associated with tungsten-containing species were detected, which also suggests their high dispersion. In contrast, in materials with a higher content of tungsten, other diffraction peaks become noticeable besides the ones associated with metallic cobalt. All additional diffraction peaks of the XRD pattern of CoW@C-0.25 correspond to the mixed metal oxide CoWO_4_ (PDF code 00-015-0867), with the exception of a peak at a 2θ value of 47.3°, which could be attributed to the (004) plane of the triclinic WO_3_ phase (PDF code 00-020-1323). Further increase of the tungstate salt in the catalyst preparation produced the formation of the segregated phase of WO_3_ to a higher extent, as revealed by the XRD pattern of the bimetallic material CoW@C-0.50.

**Figure 2:**
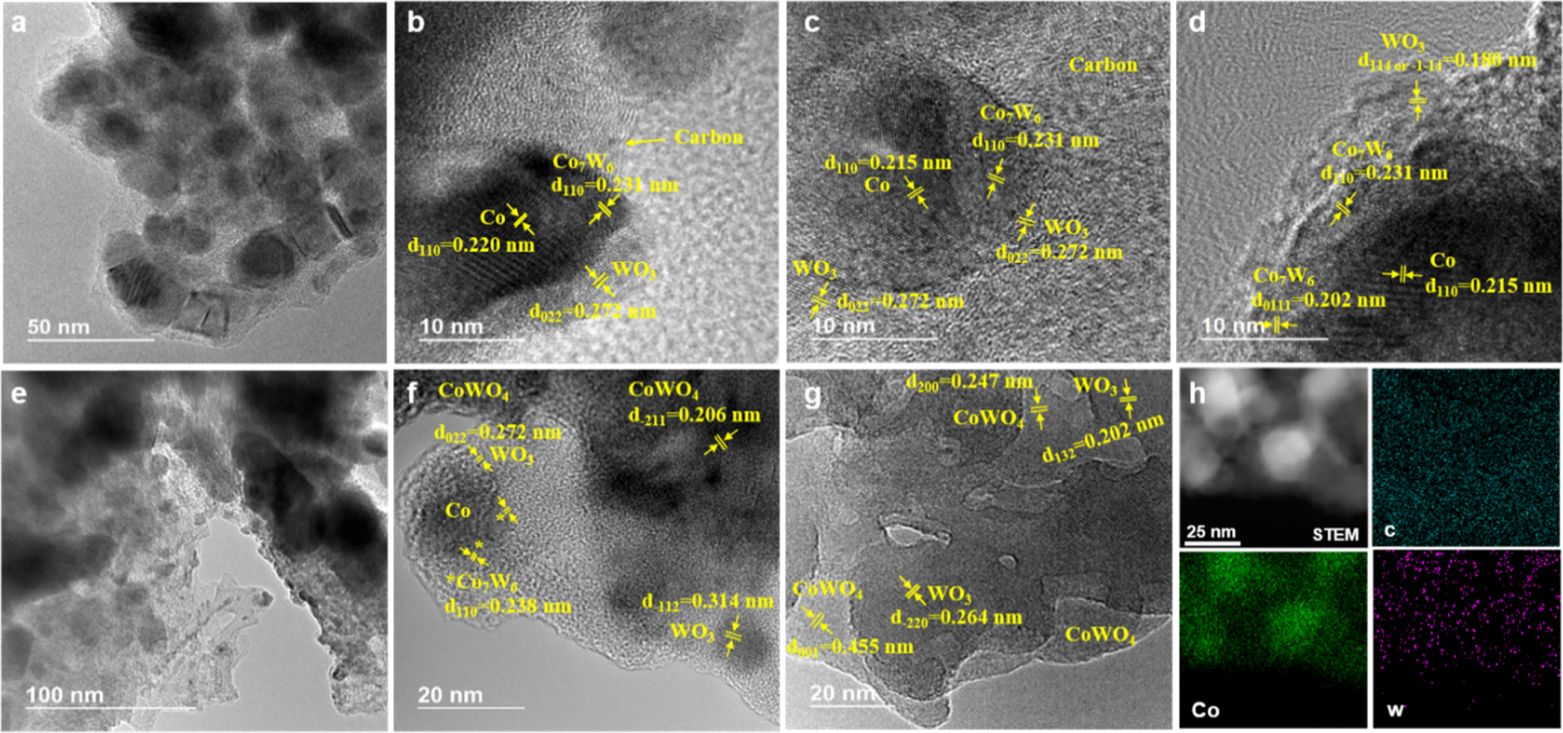
HRTEM images of catalysts (a–d) CoW@C-0.05, (e, f) CoW@C-0.25, and (g) CoW@C-0.50. (h) High-angle annular dark-field scanning transmission electron microscopy (HAADF-STEM) image of the catalyst CoW@C-0.05 and elemental mapping of C, Co, and W.

The morphology and elemental distribution of the bimetallic CoW@C and the monometallic Co@C materials were investigated by high-resolution TEM (HRTEM) and EDS elemental mapping (Figure 2; see also Figures S1–S4). CoW@C-0.05 is a nanoparticulate material with particle sizes ranging from ca. 10 to ca. 40 nm (Figure 2a–d). The NPs are embedded in a carbon matrix derived from the thermal decomposition of glucose. The crystal lattice fringes with a spacing of 0.215 nm corresponding to the (100) and (111) planes of the hexagonal Co phase can be inferred in the core of these NPs. Interestingly, other interlayer distances of 0.231 and 0.202 nm, similar to those of the (110) and (0111) planes, respectively, of the alloyed structure Co_7_W_6_, could also be found on the upper surface of these NPs. Moreover, different lattice fringes with spacings of 0.272 and 0.180 nm, which could be attributed to the (022), and either to the (114) or the (1̅1̅4) planes, respectively, of a segregated WO_3_ phase were also detected as small patches on the surface of these NPs. These results suggest that CoW@C-0.05 is composed of bimetallic NPs, partially embedded in a carbon matrix, that display a multishell core–shell structure with a metallic Co core, a W-enriched shell involving Co_7_W_6_ alloyed structures, and small WO_3_ patches partially covering the surface of these NPs. This is consistent with the EDS elemental mapping (Figure 2h) and, more precisely, with the quantitative EDS analyses from selected regions of these NPs (Figure S1) that confirm their bimetallic distribution with a major concentration of W species on the surface.

The HRTEM micrographs of the material CoW@C-0.25 revealed a more heterogeneous size distribution of bigger (up to 80 nm) and more agglomerated NPs of different nature, all of them embedded in a carbon matrix (Figures 2e,f and S2). In addition to bimetallic CoW NPs, WO_3_ NPs of ca. 10 nm size, and the mixed metal oxide CoWO_4_ in the form of larger NPs could also be detected. In contrast, the material with the highest content of tungsten, CoW@C-0.50, displayed a different morphology, where NPs, although present, were hardly found (Figures 2g and S3). This material is mainly composed of interlaced phases that display characteristic lattice-fringe spacings of 0.264 and 0.202 nm, respectively, associated with the (2̅00) and (132) planes of WO_3_, as well as of 0.455 and 0.247 nm associated with the (001) and (200) planes, respectively, of CoWO_4_.

Further evidence on the different nature of the non-metallic phase composition for catalysts CoW@C synthesized with different W/Co molar ratios was obtained by the temperature-programmed reduction under hydrogen (H_2_-temperature-programmed reduction (H_2_-TPR); see Figure S5). Reduction profiles for catalysts Co@C and CoW@C-0.05 do not differ significantly and show two different H_2_-uptake peaks, the first one at ∼180 to 200 °C and a second one that finalizes around 400–500 °C. These reduction events are associated with the sequential reduction of Co_3_O_4_ (a phase also detected by Raman spectroscopy, as shown in Figure S6) to CoO, and to metallic Co, respectively.^106^ Interestingly, the reduction profile of the catalyst containing tungsten doping species (CoW@C-0.05) is slightly shifted to lower temperatures, thus indicating that their presence influences the cobalt oxide reduction. Moreover, both catalysts display a broad H_2_-uptake peak centered at temperatures around 550–625 °C, which is typically associated with the gasification of carbon and the reduction of surface oxygenated groups present on the carbon surface.^107−109^ In addition, for the catalyst CoW@C-0.05, a broad shoulder attributed to the reduction of tungsten oxide species can also be distinguished at higher temperatures between 700 and 800 °C.^110^ The H_2_-TPR profile of the catalyst CoW@C-0.25 is dominated by the presence of a peak associated with the reduction of Co^2+^ species (at ∼330 °C) together with the broad peaks related to carbon and the reduction of tungsten oxide species. In contrast, the highest H_2_ consumption for the catalyst CoW@C-0.50 takes place in the reduction zone between 700 and 800 °C due to the presence of the tungsten oxide species to a higher extent.

### Catalytic Performance of Bimetallic CoW@C Materials for the Hydrogenation of Quinoline

The hydrogenation of quinoline (**1a**) to its partially reduced product 1,2,3,4-tetrahydroquinoline (**2a**) was used as a model reaction to compare the catalytic activity of the bimetallic CoW@C and the monometallic Co@C materials. Initial hydrogenation experiments were performed at 100 °C, 8 bar of H_2_, and using toluene as a solvent. Based on the H_2_-TPR results, and according to our previous work on related Co NPs in which the surface of the metal NPs was partially oxidized by contact with air,^101,102^ a catalyst pre-activation treatment (170 °C, 2 h, 10 bar H_2_) in the same autoclave was performed before the catalytic reaction. In the absence of this pre-activation treatment, an induction period associated with the in-situ reduction of the oxidized surface under catalytic reaction conditions was observed (Figure S8), thus suggesting that the presence of metallic species on the surface is crucial for the reaction to be accomplished.

**Figure 3:**
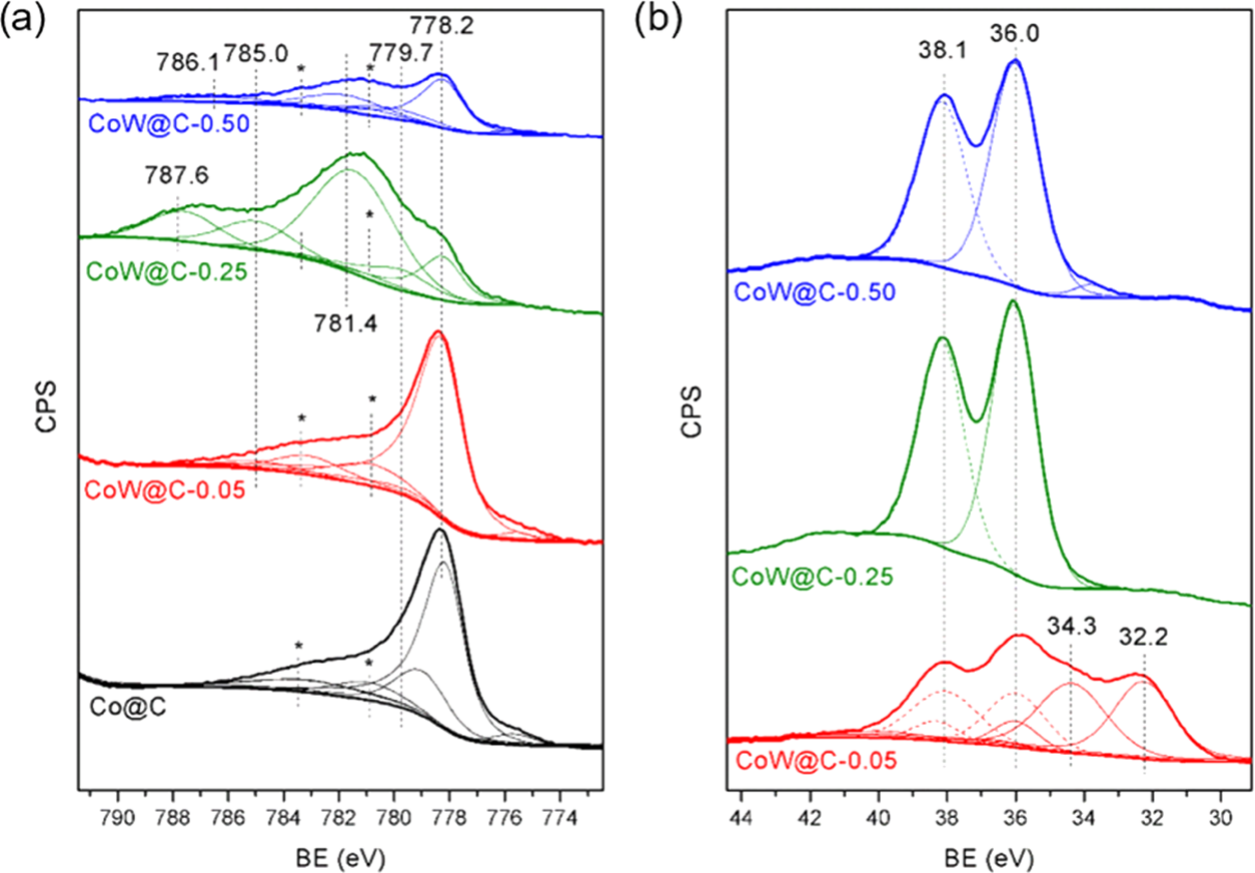
Co 2p_3/2_ (a) and W 4f (b) regions of the XPS spectra of the in-situ reduced catalysts CoW@C and Co@C.

The surface composition and oxidation state of the in-situ reduced catalysts were investigated by X-ray photoelectron spectroscopy (XPS). The XPS of Co 2p_3/2_ are shown in Figure 3a and their associated components are included in Table 1. From the surface atomic ratio, it is observed that the content of metal species on the surface decreases by increasing the tungsten loading in the catalyst preparation due to the formation of metal oxides to a higher extent and/or to a more efficient embedding of these species in the carbon matrix. In all materials, a peak at 778.2 eV due to metallic cobalt together with two plasmon loss peaks at ∼3.0 and 5.0 eV above the main peak is observed. Other components at higher binding energy (BE) associated with the oxidized cobalt species are also present (779.7–786.2 eV) in combination with the associated satellite peaks, which were also included in the deconvolution and fitting in spite of the fact that their identification was difficult because of their overlapping with other peaks. More specifically, the peak at BE of 779.7 eV corresponds to the Co^3+^ (i.e., Co_3_O_4_) species and it is observed as a minority phase. In addition, the peak at BE of 781.4 eV has been related to the highly ionic Co^2+^ type species^111^ that can be correlated to CoWO_4_, which appears in catalysts CoW@C-0.25 and CoW@C-0.50, in good agreement with the XRD and HRTEM characterization. The presence of oxidized and metallic cobalt species in all materials is also confirmed in the CoL3VV auger spectra (Figure S7), where the main peak at 773 eV is associated with metallic cobalt, while the shoulder at 767.0 eV corresponds to the oxidized cobalt species.^112^ However, from the Co XPS BE, it is difficult to differentiate between the metallic cobalt and CoW alloyed species, whose BE appears in both cases at around 778.2 eV.^113^

**Table 1:** XPS Binding Energy (BE) Values and Surface Concentration for the In Situ Reduced Catalysts CoW@C and Co@C

	Co 2p_3/2_ (BE (eV))		W 4f_7/2_ (BE (eV))			c
sample	Co^0^ (%)	Co^*n*+^ (%)	W^0^ (%)	W^4+^ (%)	W^6+^ (%)	Co/W/O/C^c^
Co@C	778.2 780.9^a^; 783.2^a^ (74.8%)	779.1; 783.2^b^ (25.2%)				14.5:--:15.2:70.4
CoW@C-0.05	778.2 780.8^a^; 783.1^a^ (90.1%)	779.7; 785.0^b^ (9.9%)	32.2 (41.1%)	34.4 (43.3%)	36.0 (15.6%)	8.2:2.9:29.2:59.7
CoW@C-0.25	778.2 780.9^a^; 783.1^a^ (13.5%)	779.7; 785.0^b^ 781.4; 787.6^b^ (86.5%)			36.0 (100%)	5.7:4.1:29.2:61.0
CoW@C-0.50	778.2 780.9^a^; 783.1^a^ (60.1%)	780.1; 785.0^b^ 781.8; 786.1^b^ (39.9%)			36.0 (100%)	2.0:3.5:20.0:74.4

a Plasmon loss peaks of cobalt.

b Satellite peak of oxidized cobalt.

c Surface atomic ratio.

Regarding the tungsten species, the XPS W 4f core level spectra (Figure 3b and Table 1) of both CoW@C-0.25 and CoW@C-0.50 materials exclusively show two peaks with BE of 36.0 and 38.1 eV, which are associated with the characteristic spin–orbit splitting of 4f_7/2_ and 4f_5/2_, respectively, and denote the presence of W^6+^ species, indicating that the tungsten species were in the forms WO_3_ and CoWO_4_ on the surface of these catalysts, while no peaks associated to metallic tungsten were observed. In contrast, the W 4f core level spectrum of CoW@C-0.05 after deconvolution and fitting also displays two additional doublet peaks at a lower BE due to the presence of W^4+^ (34.4 and 38.1 eV) and W^0^ (32.2 and 36.0 eV) species.^114^ Interestingly, the BE ascribed to W^0^ is slightly shifted to higher BE (32.2 eV) compared to the literature data (31.6 eV), which can tentatively be ascribed to the formation of CoW alloyed species.

**Figure 4:**
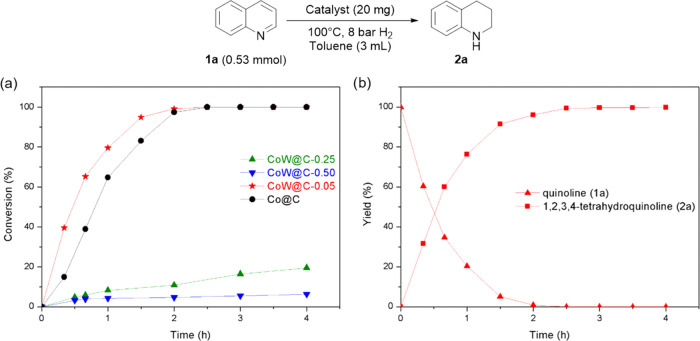
(a) Catalytic performances of CoW@C and Co@C for the hydrogenation of quinoline (**1a**). (b) Yields of **1a** and 1,2,3,4-tetrahydroquinoline (**2a**) vs time for the catalyst CoW@C-0.05.

The catalytic performance of the bimetallic CoW@C and the monometallic Co@C materials for the hydrogenation of **1a** is shown in Figure 4. The catalyst CoW@C-0.05 containing the surface CoW alloyed species displayed higher activity (higher than twice the initial reaction rate) than the monometallic catalyst Co@C, while a further increase of the tungsten content in the catalyst preparation led to detrimental results. This sharp decrease in the catalytic activity observed for catalysts CoW@C-0.25 and CoW@C-0.50 can be ascribed to the formation of both the bimetallic CoWO_4_ and the monometallic WO_3_ oxides as separate phases, which displayed a negligible conversion of **1a** when they were pre-activated and used as catalysts under otherwise the same conditions (Figure S9). When the tungsten doping content was further reduced in the catalyst preparation, the obtained catalyst (CoW@C-0.025) also displayed a slightly lower activity than the catalyst CoW@C-0.05 (Figure S9).

**Figure 5:**
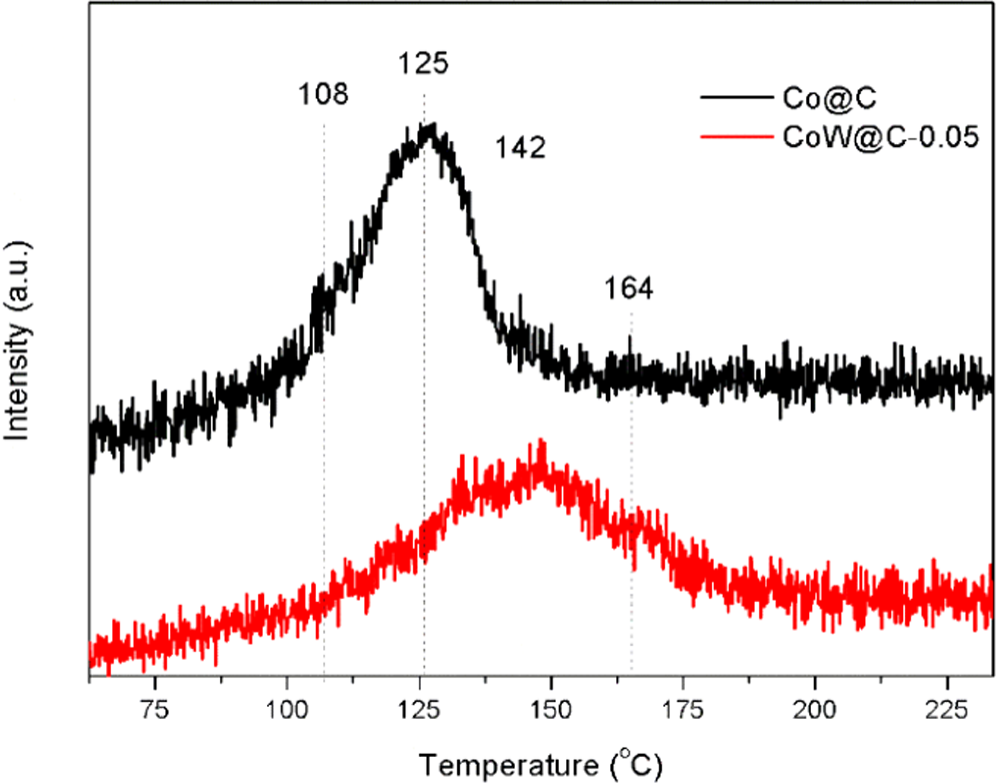
Temperature-programmed desorption (TPD) mass spectrometry experiments on CoW@C and Co@C.

In terms of regioselectivity, CoW@C-0.05 afforded a quantitative yield (99%) of **2a** after full conversion of **1a**, with no formation of other byproducts even after longer reaction times (Figure 4b). In contrast, in the presence of the catalyst with lower tungsten doping content (CoW@C-0.025) and the monometallic Co@C material, traces (<2 and <5%, respectively) of 5,6,7,8-tetrahydroquinoline (**3a**) as byproduct were also formed by the hydrogenation of the N-free aromatic ring of **1a** (Figure S9). Assuming that the electronic structure of Co is modified by charge transfer with the tungsten doping metal,^10,115^ a feasible reason of full selectivity for the catalyst CoW@C-0.05 could be ascribed to the most acidic (i.e., electronegative) nature of the tungsten species, which could hinder to a higher extent the flat adsorption of **1a** on the catalyst surface through the higher interaction with the electron-donating N-heteroaromatic ring.^96,116−118^ To get further insights on this assumption, the interaction strengths of **1a** with catalysts CoW@C-0.05 and Co@C were investigated by analyzing their desorption profiles at increasing temperatures in the temperature-programmed desorption (TPD) setup coupled with a mass spectrometer analyzer (see the Experimental Section for more details). As shown in Figure 5, while the interaction strengths of the monometallic catalyst Co@C are characterized by desorption temperatures of 108 and 125 °C, the TPD-mass spectra of the catalyst CoW@C-0.05 also display higher desorption temperatures of 142 and 164 °C, thus revealing that quinoline (**1a**) interacts stronger on the surface of the bimetallic catalyst CoW@C-0.05 than on the monometallic catalyst.

**Figure 6:**
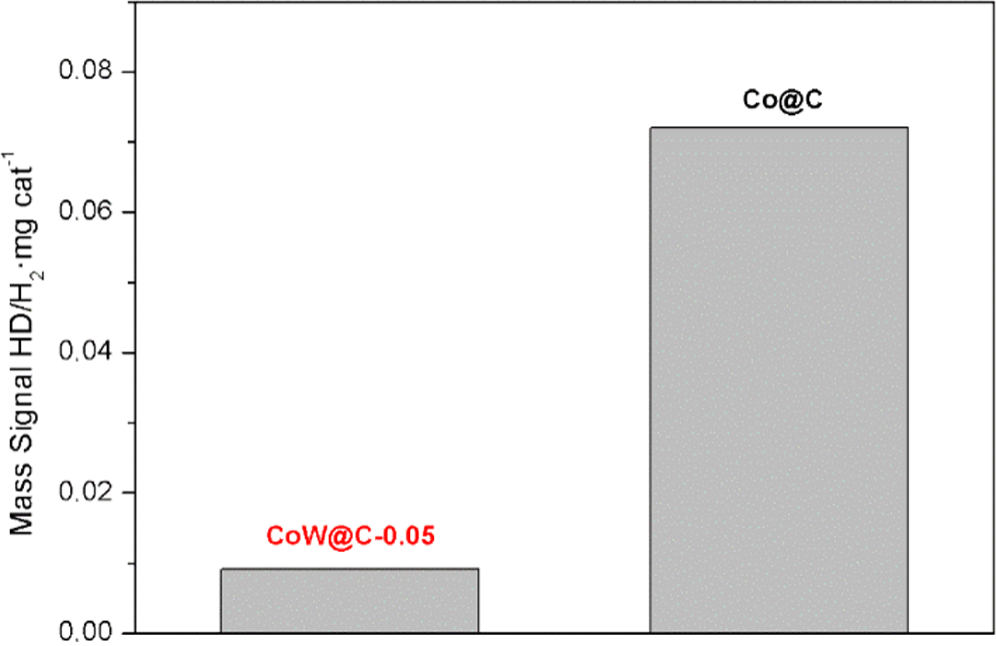
H_2_–D_2_ exchange experiments on the pre-activated catalysts CoW@C-0.05 and Co@C.

Next, H_2_–D_2_ exchange experiments were performed over the pre-activated catalysts CoW@C-0.05 and Co@C to investigate the influence of the tungsten doping metal on the catalytic performance of these nanoparticles for H_2_ activation. As shown in Figure 6, the catalyst CoW@C-0.05 displays a considerably lower H_2_ dissociation rate than the monometallic Co@C. This significant difference can be explained on the basis of higher electronegativity of tungsten, which hinders the donation of d-electrons to the σ* antibonding orbital of H_2_ to weaken the H–H bond, and consequently, a significant decrease of the ion current for the HD mass signal during the H_2_–D_2_ exchange experiments over the bimetallic CoW@C-0.05 catalyst was achieved.^5,119^ This result suggests that a homolytic dissociation of H_2_ is taking place on the surface of both catalysts rather than a heterolytic cleavage.

### Kinetic Study

**Figure 7:**
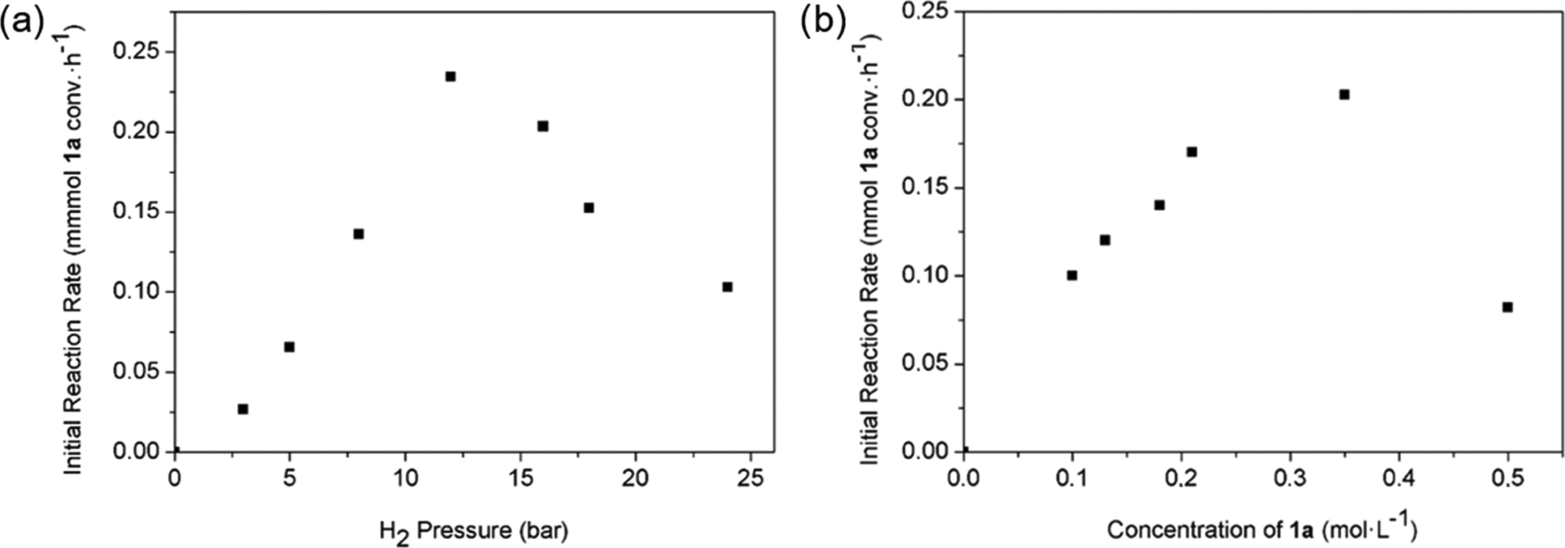
Kinetic studies of the hydrogenation of quinoline (**1a**) in the presence of CoW@C-0.05. (a) Initial reaction rate at different H_2_ pressures and 0.53 mmol of **1a**. (b) Initial reaction rate at different concentrations of **1a** at 8 bar H_2_. Reaction conditions: 10 mg CoW@C-0.05, 3 mL of toluene, 0.3 mmol of dodecane as an internal standard, 100 °C. The catalyst was pre-activated at 170 °C with 10 bar H_2_ for 2 h, and then cooled to room temperature before injecting the reaction mixture to the batch reactor.

A comparison of the catalytic activities of both CoW@C-0.05 and Co@C catalysts with the results obtained from the H_2_–D_2_ exchange and TPD-mass spectrometry experiments reveals that the most active catalyst (i.e., CoW@C-0.05) displays lower H_2_ dissociation activity and a stronger interaction with quinoline (**1a**), thus demonstrating that the activation of H_2_ cannot be the controlling step of the process, and suggests that it could be controlled by the adsorption/activation of **1a**. Therefore, to get further insights into the rate-determining step of the reaction, kinetics experiments for the hydrogenation of **1a** on CoW@C-0.05 were performed by measuring the initial reaction rates at conversion levels below 20% at different H_2_ pressures and different concentrations of **1a**, while keeping one of these two reactants constant. As shown in Figure 7, in both cases, the initial reaction rate experiences a fast increase up to a maximum, followed by a decrease with increasing the H_2_ pressure or the concentration of **1a**. According to the kinetic models developed by Hougen–Watson/Langmuir–Hinshelwood for reaction mechanisms in heterogeneous catalysis,^103,120−122^ the observed evolution trends for the initial reaction rates indicate that the rate-determining step of the reaction is not H_2_ dissociation, or adsorption, or activation of **1a**, but a hydrogenation step involving a surface reaction between the activated H_2_ (metal hydrides) and the adsorbed **1a**. These results can be described by the kinetic rate equation
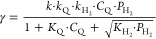
where *C*_Q_ is the concentration of **1a**, *P*_H_2__ is the H_2_ pressure, *k*_Q_, *k*_H_2__, and *k* are the kinetic constants for H_2_ dissociation, **1a** adsorption, and surface reaction, respectively, and *K*_H_2__ and *K*_Q_ are the equilibrium adsorption constants for H_2_ and **1a**.

Notably, taking into account this result and the fact that differences between the bimetallic CoW@C-0.05 and the monometallic Co@C catalysts mainly arise from the presence of CoW surface alloyed species (and non-active tungsten oxides), the superior activity of the catalyst CoW@C-0.05 can be ascribed to these alloy species, which promote a better interaction (i.e., more efficient d-orbital hybridization) between both metals (Co and W), thus likely favoring the controlling surface reaction to be more efficiently accomplished.

On the other hand, the parabolic trend observed in the kinetic experiments also suggests that both reactants (H_2_ and **1a**) share the same type of active sites on the catalyst surface. To further clarify whether the decrease of reactivity at high hydrogen pressure (>12 bar) is due to a competitive adsorption of reactants or involves an irreversible variation of the catalyst surface, a control experiment was performed. Instead of the common catalyst pre-activation treatment (at 10 bar H_2_), the catalyst CoW@C-0.05 was pre-activated at 22 bar H_2_ (pressure at which its catalytic activity for the hydrogenation of **1a** is considerably lower) and used for the hydrogenation of **1a** under standard conditions at 8 bar H_2_. Interestingly, a higher reaction rate was achieved when compared with that obtained through the common catalyst pre-activation treatment (Figure S10). This result not only confirms that a competitive adsorption/activation of H_2_ and **1a** takes place on the same active sites, but also further suggests that the active sites are the metallic species on the catalyst surface, which are formed to a higher extent by the reduction of the oxidized surface under a harsher pre-activation treatment.

### Optimization of Reaction Conditions for the Hydrogenation of Quinoline

Further optimization of the reaction conditions was carried out with the most active catalyst CoW@C-0.05. No loss of selectivity was achieved by using solvents other than toluene but it led to a lower reactivity (Table 2, entries 1–6). Remarkably, CoW@C-0.05 is also active even at a lower reaction temperature (60 °C; Table 2, entry 7). At this point, it is also worth mentioning that this catalyst allows performing the hydrogenation reaction of quinoline (**1a**) to 1,2,3,4-tetrahydroquinoline (**2a**) under milder conditions than any of the non-noble metal-based catalysts reported to date. Typically, these kinds of catalysts are only active at a relatively high temperature (>120 °C) and/or at least 20 bar of H_2_ pressure (see Table S1). In contrast, CoW@C-0.05 catalyzes this reaction at milder conditions of both temperature and H_2_ pressure (<100 °C and <8 bar H_2_, respectively). Under otherwise the same reaction conditions as that used for CoW@C-0.05, other previously reported Co-based catalysts showed lower catalytic activity, which has been determined by comparison of the initial reaction rates normalized to the mass of metal weights (see Figure S11).

**Table 2:**
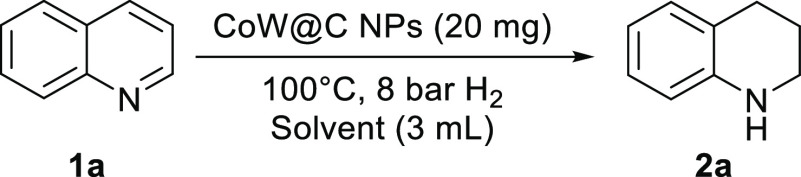
CoW@C-0.05 Catalyzed Hydrogenation of Quinoline (**1a**)^a^

entry	solvent	conversion (%)^b^	yield 2a (%)^b^
1	1,4-dioxane	93	88
2	*n*-hexane	86	83
3	2-propanol	85	84
4	THF	75	75
5	MeOH	47	43
6	toluene	>99	99
7^c^	toluene	92	88

a Reaction conditions: **1a** (0.53 mmol), catalyst (20 mg), solvent (3 mL), 6 h.

b Determined by gas chromatography (GC) using dodecane as an internal standard.

c Catalyst (40 mg), 60 °C, 24 h.

### Reusability of the Catalyst CoW@C-0.05

**Figure 8:**
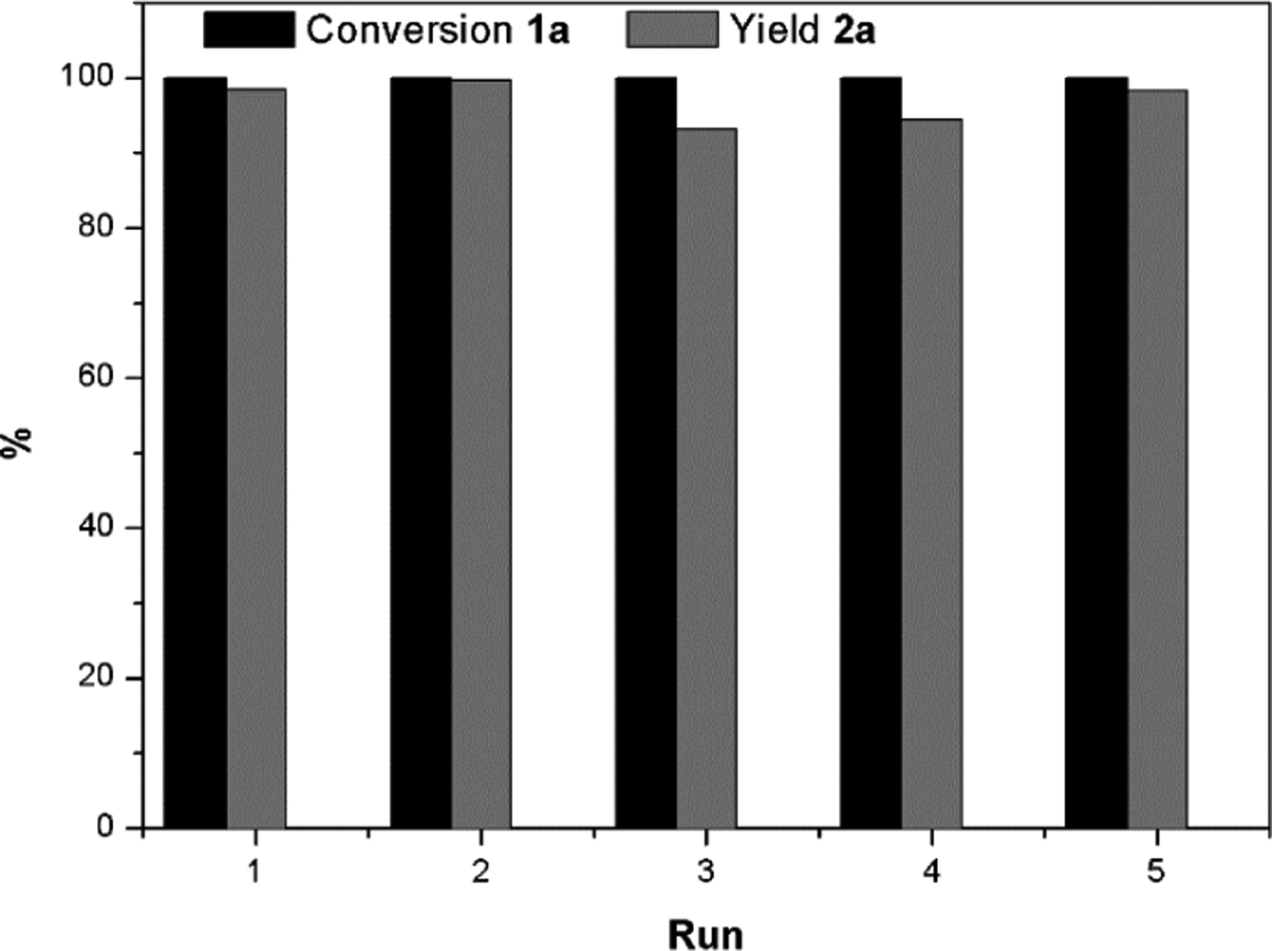
Recycling of CoW@C-0.05 for the hydrogenation of quinoline (**1a**) to 1,2,3,4-tetrahydroquinoline (**2a**). Reaction conditions: **1a** (0.53 mmol), CoW@C-0.05 (20 mg), toluene (3 mL), 6 h (run 1–3), 6.5 h (run 4), and 10 h (run 5).

**Figure 9:**
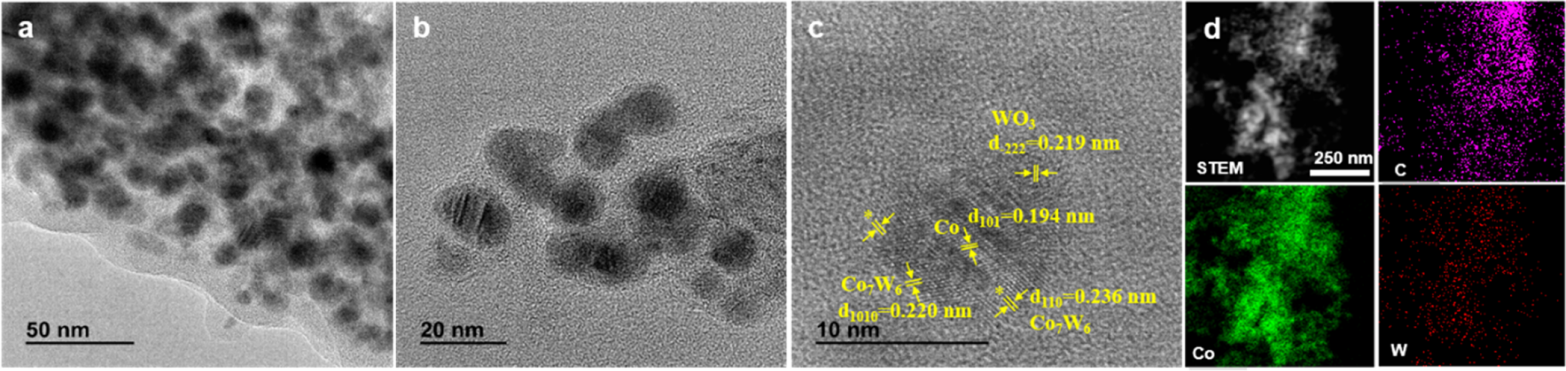
(a–c) HRTEM micrographs and (d) HAADF-STEM image and elemental mapping of Co, W and C of the recycled catalyst CoW@C-0.05 after the fifth run.

Next, we studied the catalyst recyclability for the model reaction. An important aspect of the catalyst CoW@C-0.05 is its paramagnetic character, which facilitates the catalyst separation from the liquid phase with the help of a magnetic bar. As shown in Figure 8, CoW@C-0.05 was reused for five runs achieving excellent yield of the desired product **2a** after full conversion of **1a**. It is noticeable that no metal leaching occurred in the reaction medium, as revealed by inductively coupled plasma-mass spectrometry (ICP-MS) analysis of the filtrates. The stability of this catalyst was further corroborated by XRD and TEM characterization. No additional diffraction peaks associated to oxide species were observed in the XRD pattern of the recycled catalyst (Figure S12). As in the fresh catalyst, the recycled one is formed by monodispersed NPs on thin carbon films, which still covered the NPs (Figure 9a–c). These results confirm the key protecting role of the carbon layers that confer good stability to the NPs, thus avoiding them from agglomeration and overoxidation, and therefore, allowing for a prolonged use. Furthermore, the distribution of both metals and carbon is still maintained, as revealed by HRTEM and STEM-HADDF elemental mapping (Figure 9c,d, respectively).

### Scope of the Catalyst CoW@C-0.05

Finally, we focused on the scope of the catalyst CoW@C-0.05; for that, we performed the hydrogenation of structurally diverse quinolines under the same mild reaction conditions used previously (Table 3). Quinaldine and 8-methylquinoline were smoothly hydrogenated affording the corresponding 1,2,3,4-tetrahydroquinolines in exceptional yield (Table 3, entries 1 and 2, respectively). No noticeable influence on the catalytic activity was observed when quinoline was substituted at the 2-position with a more sterically hindered phenyl group (Table 3, entry 3). The electron-rich 6-metoxy-1,2,3,4-tetrahydroquinoline could be easily prepared in excellent yield (92%) by the hydrogenation of the corresponding quinoline precursor (Table 3, entry 4). Fluoro-substituted quinolines either alone or accompanied with methyl or methoxy groups are also suitable candidates to accomplish the target selective hydrogenation reaction. In fact, the electron-deficient 6-fluoro-1,2,3,4-tetrahydroquinoline was obtained in 88% isolated yield (Table 3, entry 5), and the disubstituted quinolines were successfully converted to their corresponding 1,2,3,4-tetrahydroquinoline congeners in excellent yields (Table 3, entries 6–8). Furthermore, the more sterically hindered 8-chloro-quinaldine was hydrogenated into its pyridine-moiety hydrogenated product in 90% isolated yield (Table 3, entry 9). It is important to mention that no dehalogenated byproducts were detected in all of the above-described reactions. Interestingly, hydrogenation of quinolines bearing a redox-sensitive ester group to their 1,2,3,4-tetrahydroquinoline derivatives could also be accomplished in excellent yields (85 and 94%) without reduction of the ester group (Table 3, entries 10 and 11, respectively).

**Table 3:**
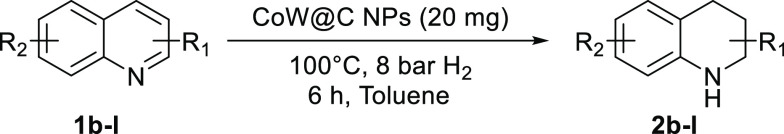

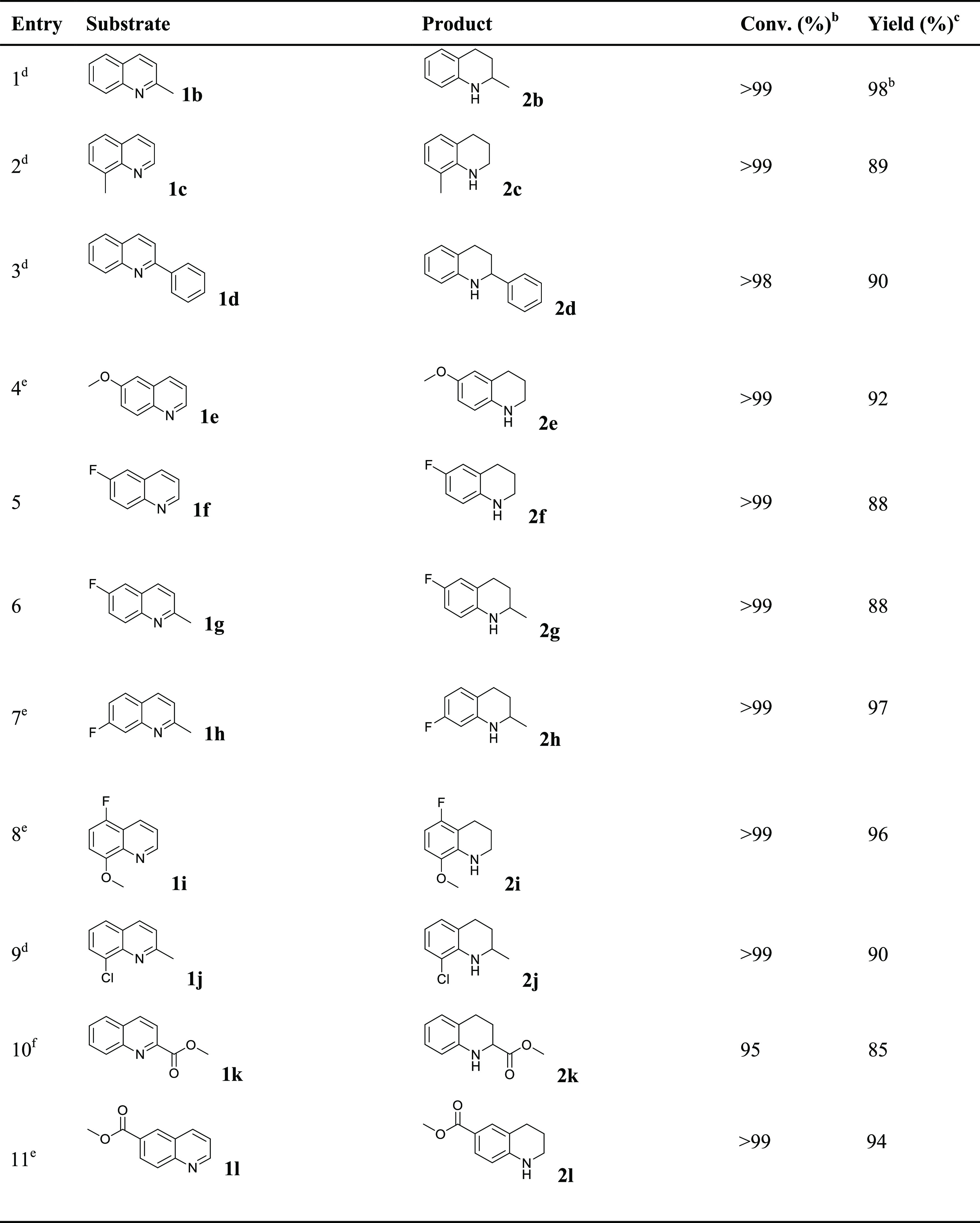
CoW@C-0.05 Catalyzed Hydrogenation of Substituted Quinolines^a^

a Reaction conditions: substrate (0.53 mmol), catalyst (20 mg), toluene (3 mL).

b Determined by GC using dodecane as an internal standard.

c Yield of the isolated product.

d 7 h.

e 7.5 h.

f 8.5 h.

## Conclusions

We have prepared bimetallic CoW@C materials by a facile hydrothermal carbon-coating methodology, followed by pyrolysis under an inert atmosphere, and applied them for the hydrogenation of quinoline (**1a**) to its partially hydrogenated product 1,2,3,4-tetrahydroquinoline (**2a**). Depending on the W/Co mole ratio used in their preparation, the synthesized materials comprise metallic Co species, including CoW alloyed structures and metal oxide phases (CoWO_4_ and WO_3_), all of them partially embedded in a carbon matrix. The higher the W/Co mole ratio, the higher segregation of oxide phases and lower the catalytic performance. In particular, the most active catalyst CoW@C-0.05 is composed of bimetallic NPs that display a multishell core–shell structure with a metallic Co core, a W-enriched shell involving Co_7_W_6_ alloyed structures, and small WO_3_ patches partially covering the surface of these NPs. Interestingly, this catalyst displays higher catalytic activity and selectivity than the monometallic Co@C catalyst. The superior catalytic performance of the catalyst CoW@C-0.05 can be attributed to the presence of the CoW alloyed species, which significantly influences its reactivity as a catalyst for the regioselective hydrogenation of **1a** to its partially hydrogenated product **2a**. More specifically, the alloyed phase promotes a better interaction of both metals (Co and W) that likely boosts the controlling surface reaction between the homolytically activated H_2_ species and the adsorbed **1a** to be more efficiently accomplished. Moreover, it has been demonstrated that the tungsten doping metals modify the Lewis acidic properties of the Co NPs, favoring the interaction of **1a** with the metallic catalyst surface (where H_2_ is also activated) to avoid the hydrogenation of the N-free aromatic ring, thus enhancing the regioselectivity of the catalytic process.

In view of the catalyst recyclability experiments, the catalyst CoW@C-0.05 has demonstrated to exhibit good stability under the reaction conditions, likely because of the protection of the thin carbon layers, which avoid from agglomeration and overoxidation. In the presence of this catalyst, a variety of functionalized quinolines, even bearing other sensitive groups such as halogens and esters, have been successfully hydrogenated to the corresponding 1,2,3,4-tetrahydroquinolines in excellent yields. It is worth mentioning the mild reaction conditions under which these selective hydrogenations take place, what makes think that this work may pave the way for designing non-noble bimetallic NPs for heterogeneous catalytic hydrogenation reactions as substitutes for precious metal-based catalysts.

## Experimental Section

### Synthesis of Catalysts CoW@C and Co@C

The bimetallic catalysts CoW@C with different W/Co mole ratios (0.025, 0.05, 0.25, and 0.50) were prepared by adapting a carbon-coating methodology previously described in our recent work.^102,103,105^ To 100 mL of a homogeneous ethylene glycol solution of Co(OAc)_2_ (4.94 g) and NaWO_4_·2H_2_O (0.23, 0.41, 2.31, or 4.62 g, respectively) at 165 °C, an aqueous solution of Na_2_CO_3_ (4.24 g, 5.62 g, 6.75 g, respectively, in 160 mL) was added drop by drop for ca. 1.5–2 h under stirring conditions. After addition, the mixture was aged at this temperature for one more hour before cooling to room temperature. Then, the formed solid was filtered, generously washed with acetone, until a dry powder was obtained. Next, one fraction of this dried solid (0.45 g) was dispersed in 20 mL of an aqueous solution of glucose (0.36 g) by ultrasonic treatment, transferred to a 35 mL stainless steel autoclave equipped with a Teflon liner, and reacted at 175 °C under static conditions for 18 h. After cooling to room temperature, the solid material was collected by filtration, washed with distilled water and acetone, and dried at 60 °C. In a final step, this solid was pyrolyzed under a N_2_ atmosphere at 600 °C for 2 h with a ramp rate of 10 °C/min.

The monometallic catalyst Co@C was prepared following the procedure described for the bimetallic CoW@C catalysts but without the addition of the NaWO_4_·2H_2_O salt.

### Catalyst Characterization

XRD analysis was carried out with a Philips X’PERT diffractometer using Cu Kα at 1.54178 Å radiation.

X-ray photoelectron spectra were collected using a SPECS spectrometer with a 150-MCD-9 detector and using a nonmonochromatic Al Kα (1486.6 eV) X-ray source. Spectra were recorded using an analyzer pass energy of 30 eV, an X-ray power of 100 W, and under an operating pressure of 10^–9^ mbar. During data processing of the XPS spectra, binding energy (BE) values were referenced to the C 1s peak (284.7 eV). Spectra treatment was performed using CASA software. The samples have been in situ pre-activated in H_2_ (10 bar) at 170 °C for 2 h, in a high-pressure catalytic reactor (HPCR) connected to the XPS equipment and transferred under vacuum for analysis.

Raman spectra were obtained from solid samples using an excitation wavelength of 785 nm in a Renishaw Raman spectrometer equipped with an Olympus microscope and a CCD detector. The laser power on the sample was ∼10 to 25 mW and a total of 20 acquisitions were taken for each spectra.

The TPD experiments were carried out in a home-made flow reactor connected to a Balzer mass spectrometer. Prior to TPD experiments, the samples were impregnated with quinoline (**1a**), following the subsequent procedure: 100 mg of samples were in situ reduced in a three-neck glass flask at 200 °C for 2 h in a H_2_ flow. Afterward, the samples were cooled in H_2_ to room temperature, and then, flushed with a N_2_ flow for 10 min. Next, 0.2 mL of the (**1a**: MeOH = 1:3) mixture was added using a syringe under a N_2_ atmosphere. After stirring for 10 min, the samples were dried under vacuum and heated at 60 °C for 1 h. For the TPD experiment, 70 mg of the impregnated sample was exposed to an Ar flow of 20 mL/min, and after 10 min stabilization at room temperature, the temperature was increased to 200 °C at a heating rate of 2 °C/min. Mass spectra were collected in a multi-ion detection mode (MID) following the fragmentation peaks: *m*/*z* = 130, 129, 102,103, 76, 78, and 79 uma. For discussion, the *m*/*z* = 78 uma fragmentation peak was used.

H_2_–D_2_ exchange experiments were performed in a flow reactor. The reaction products (H_2_, HD, D_2_) were analyzed with a mass spectrometer (Omnistar, Balzers). The Co@C and CoW@C samples were pre-activated at 200 °C for 2 h with a temperature increasing rate of 10 °C/min from room temperature to 200 °C.

The samples for HR-TEM were ultrasonically dispersed in CH_2_Cl_2_ and transferred into carbon-coated copper grids. HR-TEM images were recorded using a JEOL JEM2100F microscope operating at 200 kV. The spatial distribution of Co@C and CoW@C samples were determined using an energy-dispersive X-ray analysis (EDXA) system (Oxford Instruments) attached to a JEOL JEM2100F electronic microscope.

The GC yields were determined by a GC-flame ionization detection (GC-FID) using dodecane as an internal standard. GC-FID analyses were performed on a Bruker 430-GC System equipped with a 25 m capillary column of 5% phenylmethylsilicone. Mass determination was carried out on a GC-Mass Agilent 6890 Network equipped with the same column as the GC and a mass selective detector. ^1^H NMR and ^13^C NMR spectra of the isolated products were recorded on a Bruker AV 300 spectrometer.

### Catalytic Experiments

Hydrogenation experiments were carried out in a 12 mL stainless steel autoclave equipped with a Teflon liner, a pressure controller, and a cannula ending with an open/off valve that allows for taking out samples during the reaction. The Teflon vessel containing a stirring bar was charged with 20 mg of catalyst CoW@C (or Co@C) and introduced into the stainless steel autoclave. After sealing, the autoclave was purged by flushing three times with 10 bar H_2_, pressurized again, and kept at 170 °C for 2 h. Then, the autoclave was cooled to room temperature and carefully depressurized to 1.5–2 bar H_2_. Without opening the autoclave, a mixture of the quinoline substrate (0.53 mmol), dodecane as an internal standard (0.3 mmol) and toluene as a solvent (3 mL) were added though the incorporated cannula. Next, the H_2_ was increased to 8 bar, and the autoclave was seated into an aluminum block located on a heating plate previously set at 100 °C and 1100 rpm of stirring speed. To follow the reaction, aliquots (20 μL) were taken out from the reaction mixture for GC and GC-mass analysis at different reaction times. For catalyst recycling experiments, after the completion of the reaction, the autoclave was cooled to room temperature and depressurized. Because of the paramagnetic nature of these catalysts, they could be easily recovered with the stirring bar, thoroughly washed with fresh toluene, dried at ambient conditions, and used for the next run. When isolated yields are given, the catalytic reactions were performed in the absence of any internal standard. After the removal of the catalyst, the reaction mixture was collected with ethyl acetate and was dried under reduced pressure.

## Supporting Information Available

Extended data on the characterization of catalysts CoW@C and Co@C; additional experimental results and procedures; and characterization data of isolated products (PDF)
