# Supplementary material for: Tuning the Catalytic Performance of Cobalt Nanoparticles by Tungsten Doping for Efficient and Selective Hydrogenation of Quinolines under Mild Conditions
Source: ACS Catal. 2021 Jun 18;11(13):8197–210. doi: 10.1021/acscatal.1c01561 (PMC9131458; doi:10.1021/acscatal.1c01561)
Supplement: Supplementary file 1 — cs1c01561_si_001.pdf [file cs1c01561_si_001.pdf]

Supporting Information

Tuning the Catalytic Performance of Cobalt Nanoparticles by Tungsten Doping for Efficient and Selective Hydrogenation of Quinolines under Mild Conditions

Marta Puche,[†] Lichen Liu,[†,‡] Patricia Concepción,[†] Iván Sorribes,[,†] and Avelino Corma*[,†]*

[†]Instituto de Tecnología Química, Universitat Politècnica de València-Consejo Superior de Investigaciones Científicas, Avenida de los Naranjos s/n, 46022 Valencia, Spain.

[‡]Present address: Department of Chemistry, Tsinghua University, 100084 Beijing, China.

*E-mail: acorma@itq.upv.es
ivsorter@itq.upv.es

1. GENERAL INFORMATION

2. CHARACTERIZATION OF MATERIALS CoW@C AND Co@C

3. EXTENSION DATA FOR CATALYTIC EXPERIMENTS

4. CHARACTERIZATION DATA AND EXPERIMENTAL DETAILS OF THE ISOLATED PRODUCTS

5. REFERENCES

6. ^{1}H AND ^{13}C NMR SPECTRA OF THE ISOLATED PRODUCTS

1. GENERAL INFORMATION

Reagents were obtained from commercial sources and were used as received. ^{1}H-NMR, ^{13}C-NMR spectra of isolated products were recorded on a Bruker AV 300 spectrometer. All chemical shifts (δ) are reported in parts per million (ppm) and coupling constants (*J*) in Hz. For ^{1}H-NMR all chemical shifts are reported relative to tetramethylsilane (δ 0.0 ppm in $CDCl_3$) or *d*-solvent peaks (δ 77.16 ppm $CDCl_3$) for ^{13}C-NMR. The GC yields were determined by GC-FID using dodecane as an internal standard. GC-FID analyses were performed on a Bruker 430-GC System equipped with a 25 m capillary column of 5% phenylmethylsilicone. Mass determination was carried out on a GC-Mass Agilent 6890 Network equipped with the same column as the GC and a mass selective detector.

2. CHARACTERIZATION OF MATERIALS CoW@C AND Co@C

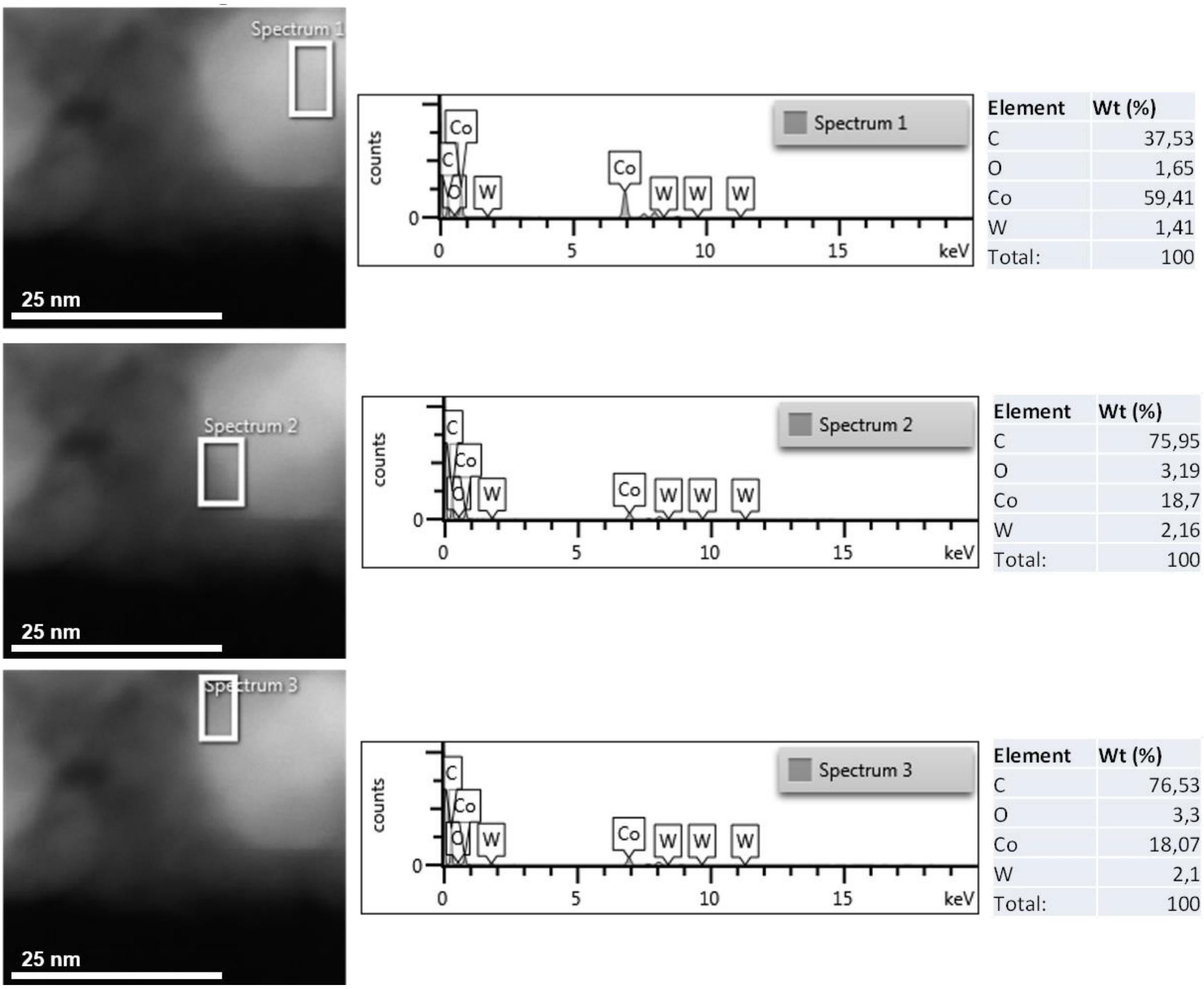

Figure S1. Area-selected quantitative EDS elemental mapping of a nanoparticle of catalyst CoW@C-0.05.

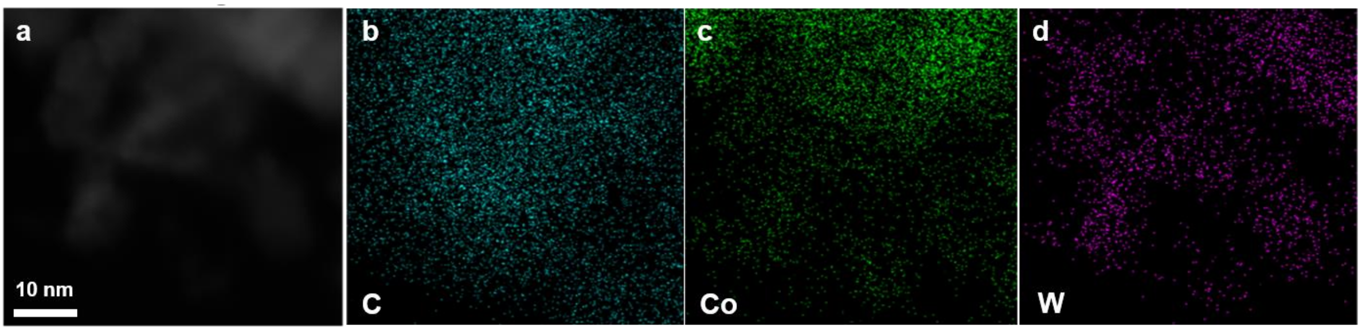

Figure S2. STEM-HADDF image (a) and elemental mapping (b-d) of C, Co and W for catalyst CoW@C-0.25.

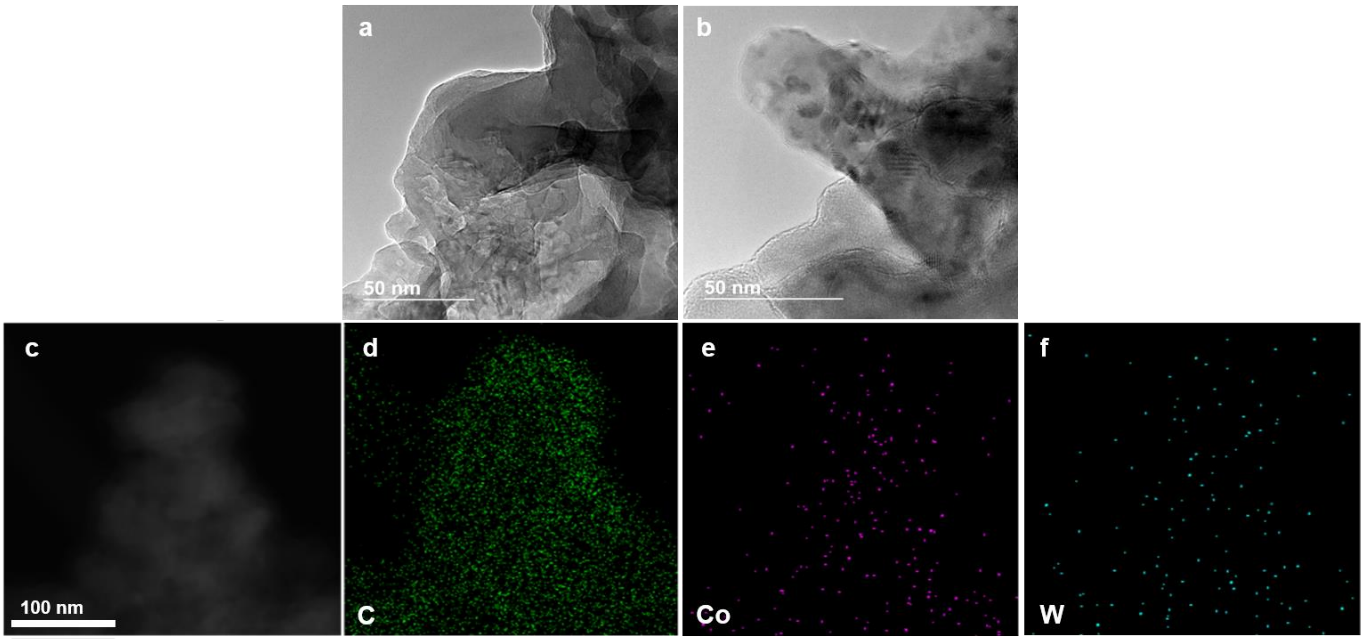

Figure S3. HRTEM (a-b), STEM-HADDF image (c), and elemental mapping of (d-f) of C, Co and W for catalyst CoW@C-0.50.

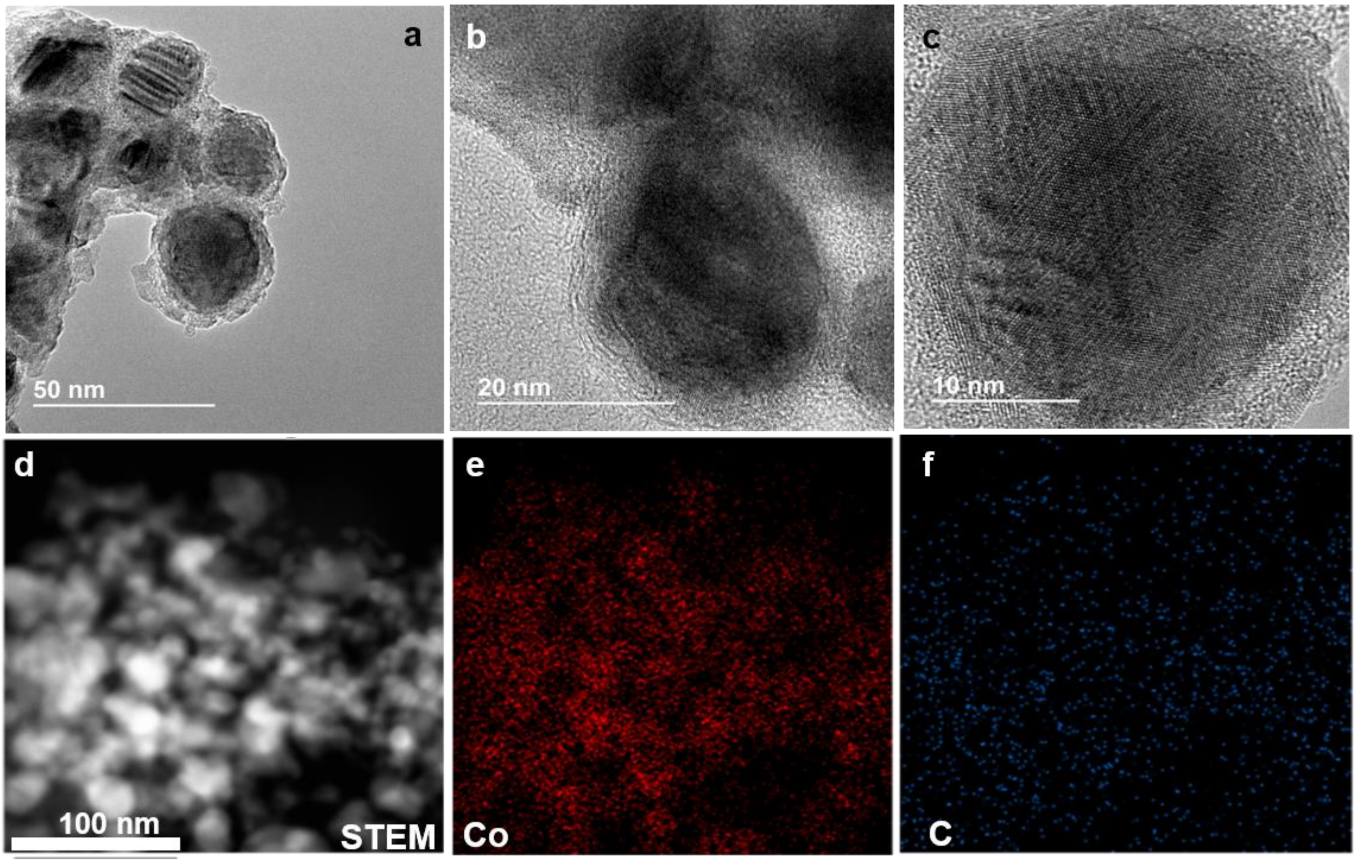

Figure S4. HRTEM (a-c), STEM-HADDF image (d), and elemental mapping of Co and C (e-f) for catalyst Co@C.

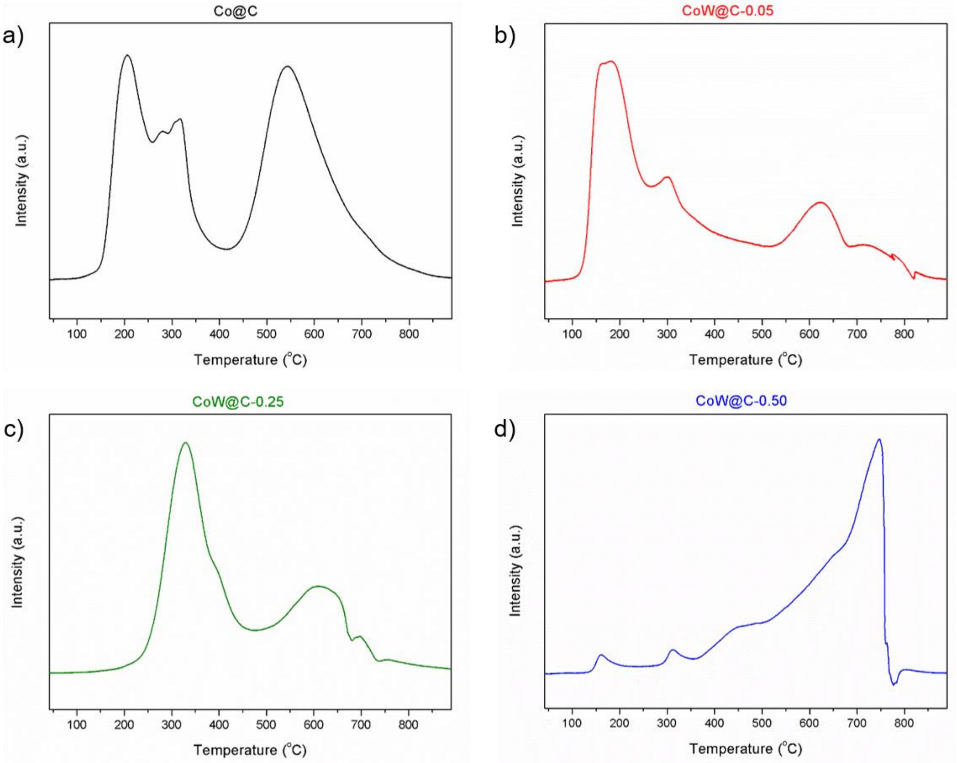

Figure S5. H_2-TPR profile of catalyst a) Co@C, b) CoW@C-0.05, c) CoW@C-0.25, and d) CoW@C-0.50.

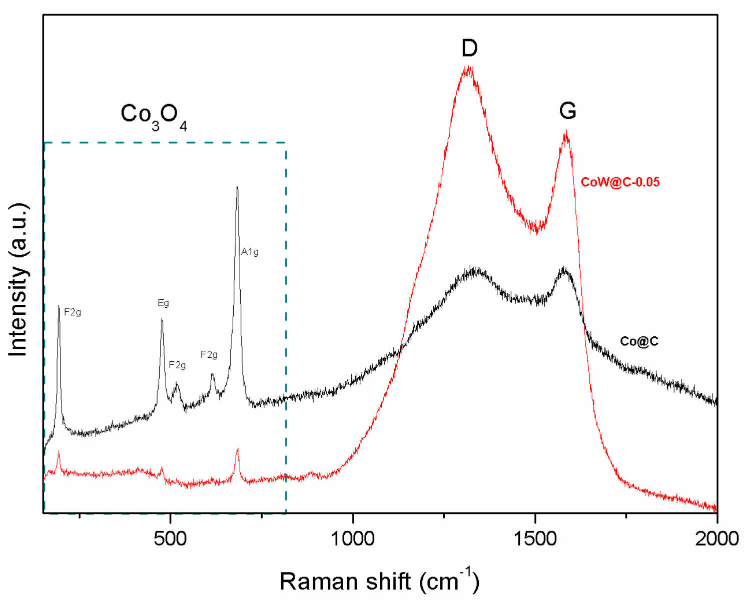

Figure S6. Raman spectra of catalysts Co@C and CoW@C-0.05.

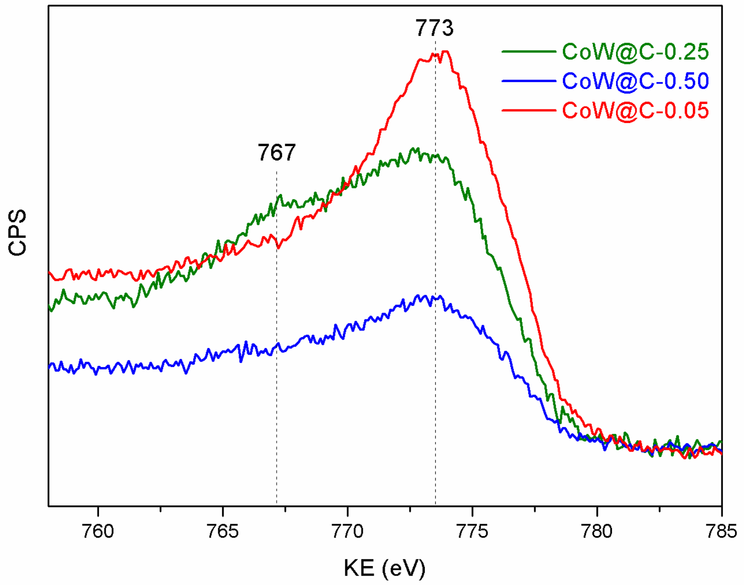

Figure S7. Co L3VV Auger spectra of bimetallic catalysts CoW@C.

3. EXTENSION DATA FOR CATALYTIC EXPERIMENTS

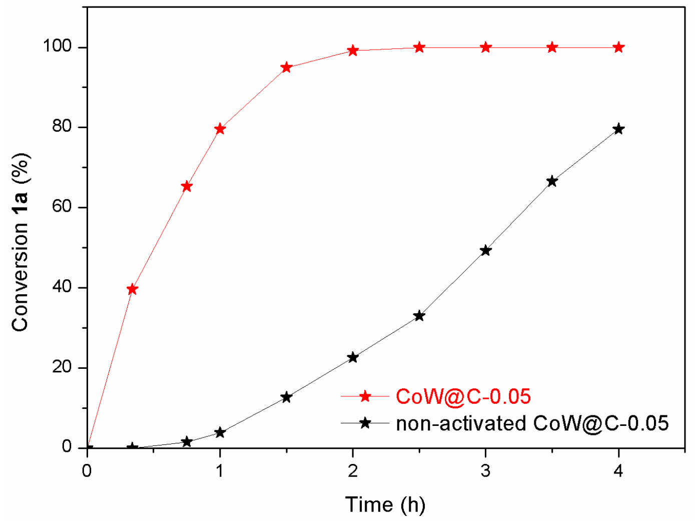

Figure S8. Comparison of the catalytic performance of catalyst CoW@C-0.05 with or without pre-activation treatment for the hydrogenation of **1a**. As it is explained in the main manuscript, the pre-activation treatment consists on keeping the catalyst in the same batch reactor for 2h, at 170 ºC and under 10 bar H_2 before catalytic reaction.

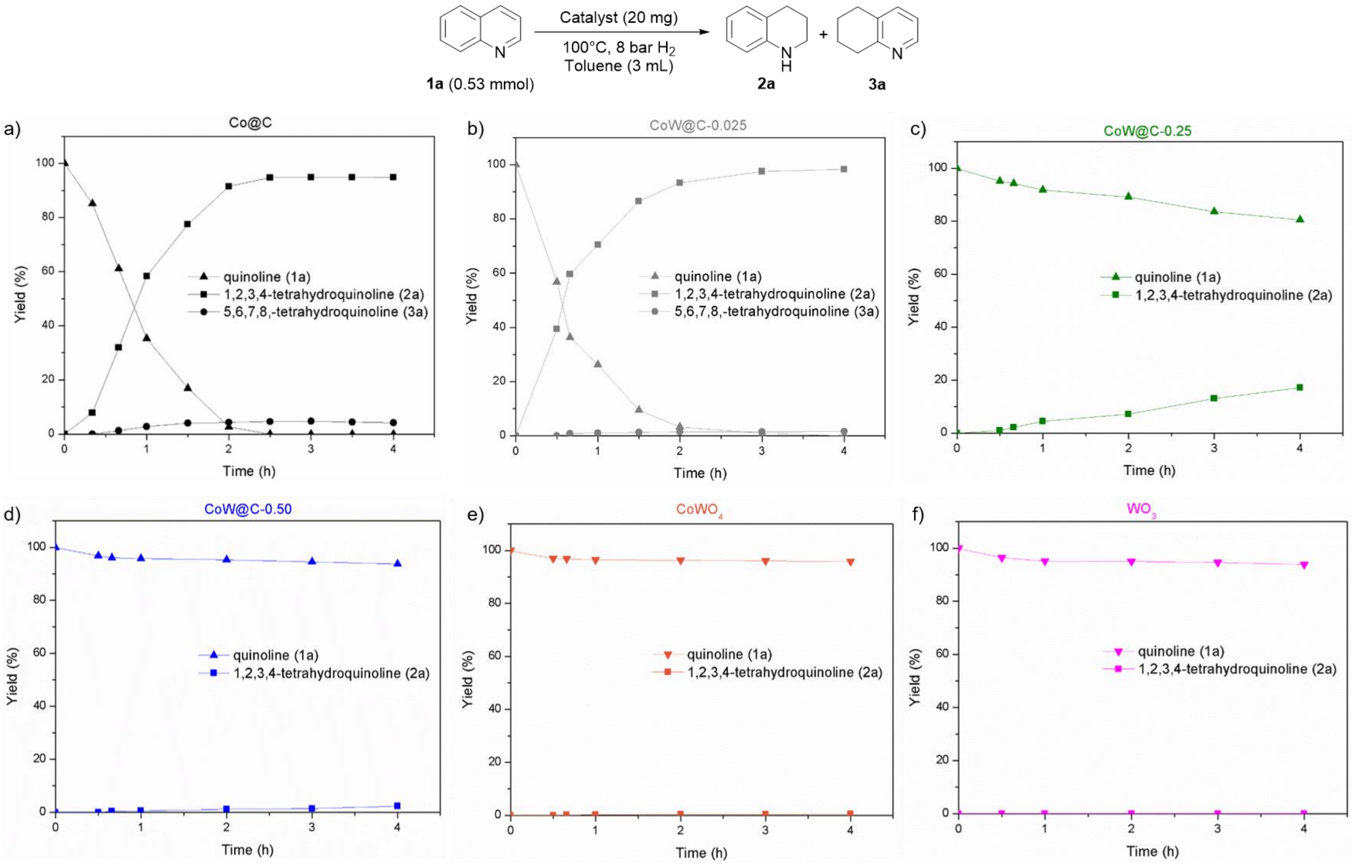

Figure S9. Concentration/time diagram for the hydrogenation of **1a** in the presence of catalyst (a) Co@C, (b) CoW@C-0.025, (c) CoW@C-0.25, (d) CoW@C-0.50, (e) $CoWO_4$, and (f) WO_3.

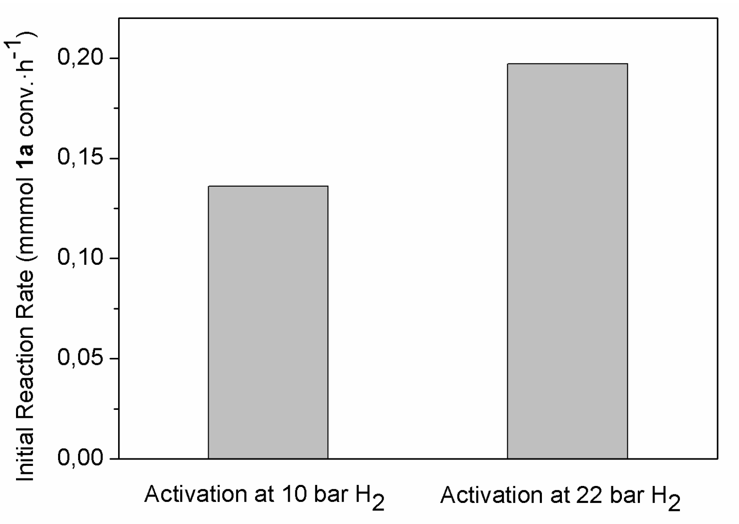

Figure S10. Comparison of the initial reaction rate for the hydrogenation of **1a** in the presence of catalyst CoW@C-0.05 pre-activated at different H_2 pressure. Reaction conditions: 10 mg CoW@C NPs, **1a** (0.53 mmol), 3 mL toluene, 0.3 mmol dodecane as an internal standard, 100 ºC, 8 bar H_2. The catalyst was pre-activated at 170 ºC with either 10 or 22 bar H_2 for 2 h, and then cooled down to room temperature before injecting the reaction mixture to the batch reactor. The initial reaction rates were calculated for conversions of **1a** below 20%.

Tables S1. Comparison between CoW@C-0.05 and heterogeneous non-noble metal catalysts previously reported for the hydrogenation on quinoline (**1a**) to tetrahydroquinoline (**2a**).

**Catalyst**	**Solvent**	**Temperature [ºC]**	**H_2 Pressure [bar]**	**Time [h]**	**Conv.(Yield) [%]**
Co_3O_4–Co/NGr@α-Al_2O_3[1]	toluene	120	20	48	>99 (98)
CoOx@CN[2]	methanol	120	30	3	>99 (>99)
Co@NGS[3]	isopropanol	140	40	24	99 (97)
Co-Mo-S[4]	toluene	120	12	12	99 (99)
Fe-N-C[5]	isopropanol	130	40	56	>99 (87)
Co NCs/NC[6]	tetrahydrofurane	120	20	6	~99 (--)
Co-SA/AC@N-CNTs-L[7]	ethanol	100	20	4	97.4 (99.1)
$CoCl_2/NaBH_4$[8]	water	130	30	17	>99 (>99)
Cu/Al_2O_3[9]	toluene	80	40	24	>99 (>99)
CoCu[10]	tetrahydrofurane	60	40	15	97 (>99)
Cu/TiO_2[11]	mesitylene	150	1	6	95 (95)
$Ni_{31}Si_{12}/Ni_2Si@SiO_2$[12]	methanol/water	120	30	16	>99 (92)
$Co_{0.14}/N_{0.11}C$[13]	--	120	30	56	>99 (>99)
Co-Phen@C[14]	Toluene	120	>30	72	-- (98%)
CoW@C-0.05 (this work)	toluene	100	8	6	>99 (99)

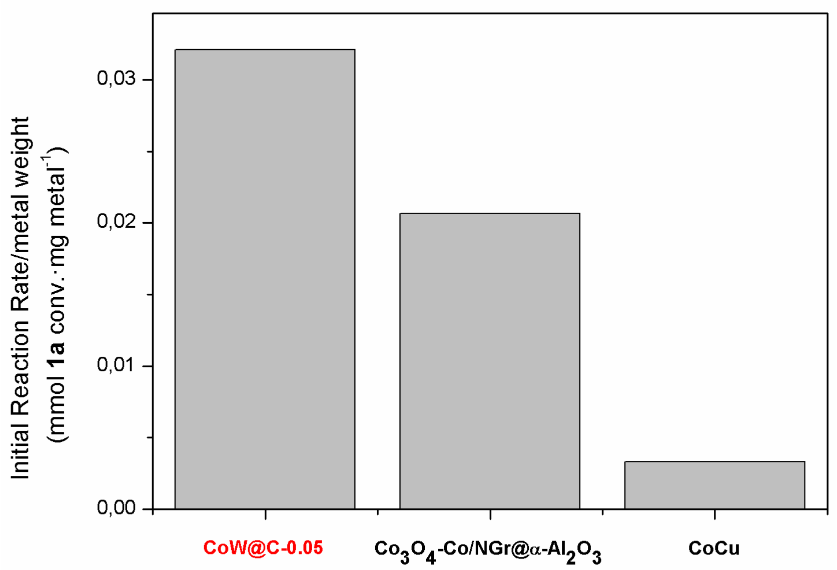

Figure S11. Comparison of the metal mass activity of non-noble metal NPs prepared from different methods. Co_3O_4–Co/NGr@α-Al_2O_3[1] and CoCu[10] catalyst were prepared according to the methods reported on the literature. Reaction conditions: catalyst (20 mg), **1a** (0.53 mmol), 8 bar H_2, 3 mL toluene, 0.3 mmol dodecane as an internal standard, 100 ºC. Catalysts were pre-activated at 170 ºC with 10 bar H_2 for 2 h, and then cooled down to room temperature before injecting the reaction mixture to the batch reactor. The initial reaction rates were calculated for conversions of **1a** below 20%.

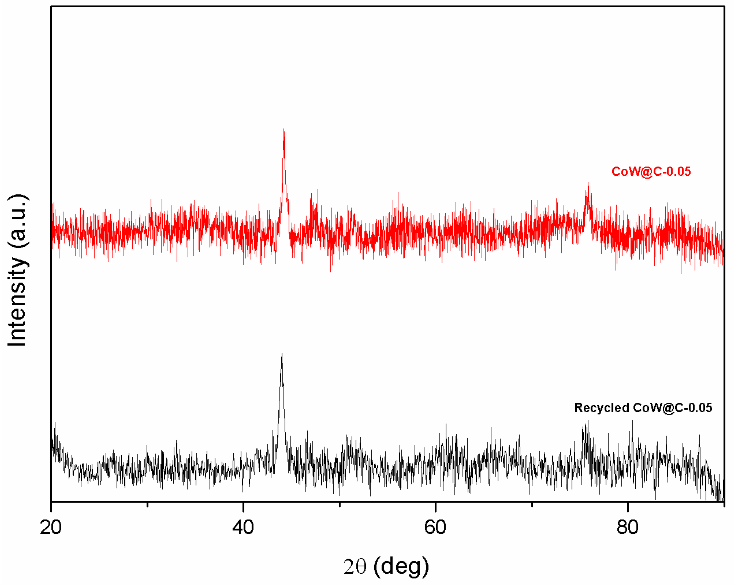

Figure S12. XRD patterns of the fresh and reused CoW@C-0.05 catalyst after the fifth run.

4. CHARACTERIZATION DATA AND EXPERIMENTAL DETAILS OF THE ISOLATED PRODUCTS

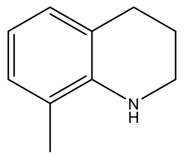

8-methyl-1,2,3,4-tetrahydroquinoline (2c): Yield: 89% (The NMR spectra are consistent with the reported data). ^{1}H NMR (300 MHz, $CDCl_3$) δ 6.97 – 6.86 (m, 2H), 6.61 (td, *J* = 7.4, 2.1 Hz, 1H), 3.61 (br, 1H), 3.47 – 3.37 (m, 2H), 2.84 (t, *J* = 6.4 Hz, 2H), 2.12 (d, *J* = 2.3 Hz, 3H), 1.99 (ddt, *J* = 11.1, 8.7, 5.2 Hz, 2H). 13C NMR (75 MHz, $CDCl_3$) δ 142.80, 127.94, 127.47, 121.26, 120.95, 116.49, 42.45, 27.40, 22.27, 17.25. MS (EI): *m/z* (rel. int.) 147.

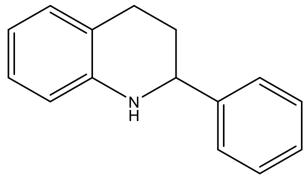

2-phenyl-1,2,3,4-tetrahydroquinoline (2d): Yield: 90% (The NMR spectra are consistent with the reported data).[1, 4, 15] ^{1}H NMR (300 MHz, $CDCl_3$) δ 7.61 – 7.34 (m, 5H), 7.13 (t, *J* = 7.5 Hz, 2H), 6.78 (t, *J* = 7.4 Hz, 1H), 6.63 (d, *J* = 7.9 Hz, 1H), 4.53 (dd, *J* = 9.3, 3.4 Hz, 1H), 4.10 (br, 1H), 3.19 – 2.95 (m, 1H), 2.84 (dt, *J* = 16.4, 4.9 Hz, 1H), 2.30 – 1.89 (m, 2H). ^{13}C NMR (75 MHz, $CDCl_3$) δ 144.89, 144.77, 129.33, 128.60, 127.46, 126.94, 126.59, 120.88, 117.19, 114.04, 56.26, 31.02, 26.40. MS (EI): *m/z* (rel. int.) 209.

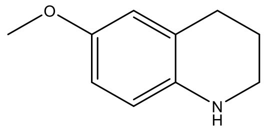

6-methoxy-1,2,3,4-tetrahydroquinoline (2e): Yield 92% (The NMR spectra are consistent with the reported data).[1, 4, 15a, 16] ^{1}H NMR (300 MHz, $CDCl_3$) δ 6.64 – 6.53 (m, 2H), 6.44 (d, *J* = 8.4 Hz, 1H), 3.72 (s, 3H), 3.55 (br, 1H), 3.29 – 3.19 (m, 2H), 2.75 (t, *J* = 6.5 Hz, 2H), 1.99 – 1.85 (m, 2H). ^{13}C NMR (75 MHz, $CDCl_3$) δ 151.86, 138.82, 122.88, 115.62, 114.88, 112.91, 55.78, 42.33, 27.17, 22.43. MS (EI): *m/z* (rel. int.) 163.

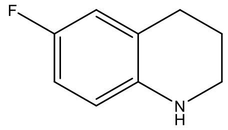

6-fluoro-1,2,3,4-tetrahydroquinoline (2f): Yield 88% (The NMR spectra are consistent with the reported data).[4, 17] ^{1}H NMR (300 MHz, $CDCl_3$) δ 6.75 – 6.62 (m, 2H), 6.40 (dd, *J* = 9.5, 4.9 Hz, 1H), 3.54 (br, 1H), 3.32 – 3.22 (m, 2H), 2.75 (t, *J* = 6.5 Hz, 2H), 1.99 – 1.85 (m, 2H). ^{13}C NMR (75 MHz, $CDCl_3$) δ [157.14, 154.03 (d, $^{1}J_{C-F}$ = 234.6 Hz)], [141.06, 141.04 (d, $^{4}J_{C-F}$ = 1.8 Hz)], [122.95, 122.86 (d, $^{3}J_{C-F}$ = 6.7

Hz)], [115.87, 115.58 (d, $^{2}J_{C\text{-}F}$ = 21.6 Hz)], [115.08, 114.98 (d, $^{3}J_{C\text{-}F}$ = 7.5 Hz)], [113.46, 113.16 (d, $^{2}J_{C\text{-}F}$ = 22.4 Hz)], 42.21, [27.16, 27.14 (d, $^{4}J_{C\text{-}F}$ = 1.3 Hz)], 22.12. MS (EI): *m/z* (rel. int.) 151.

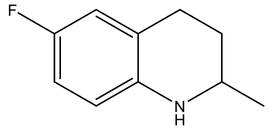

6-fluoro-2-methyl-1,2,3,4-tetrahydroquinoline (2g): Yield 88% (The NMR spectra are consistent with the reported data).[1, 4, 15a, 18] ^{1}H NMR (300 MHz, $CDCl_3$) δ 6.76 – 6.63 (m, 2H), 6.41 (ddd, *J* = 10.9, 5.6, 2.8 Hz, 1H), 3.52 (br, 1H), 3.36 (ddt, *J* = 12.5, 6.3, 3.2 Hz, 1H), 2.93 – 2.63 (m, 2H), 1.93 (ddt, *J* = 12.3, 6.0, 3.1 Hz, 1H), 1.58 (dddd, *J* = 13.0, 11.6, 9.9, 5.6 Hz, 1H), 1.22 (dd, *J* = 6.4, 2.1 Hz, 3H). ^{13}C NMR (75 MHz, $CDCl_3$) δ [157.15, 154.05 (d, $^{1}J_{C\text{-}F}$ = 234.5 Hz)], [141.10, 141.08 (d, $^{4}J_{C\text{-}F}$ = 1.9 Hz)], [122.61, 122.52 (d, $^{3}J_{C\text{-}F}$ = 6.7 Hz)], [115.61, 115.33 (d, $^{2}J_{C\text{-}F}$ = 21.6 Hz)], [114.86, 114.76 (d, $^{3}J_{C\text{-}F}$ = 7.6 Hz)], [113.39, 113.09 (d, $^{2}J_{C\text{-}F}$ = 22.4 Hz)], 47.41, 29.99, 26.80, 22.57. MS (EI): *m/z* (rel. int.) 165.

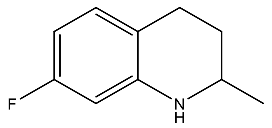

7-fluoro-2-methyl-1,2,3,4-tetrahydroquinoline (2h): Yield 97% (The NMR spectra are consistent with the reported data).[1, 4, 15a, 18] ^{1}H NMR (300 MHz, $CDCl_3$) δ 6.92 – 6.81 (m, 1H), 6.29 (td, *J* = 8.5, 2.6 Hz, 1H), 6.16 (dd, *J* = 10.9, 2.6 Hz, 1H), 3.77 (br, 1H), 3.40 (dqd, *J* = 9.4, 6.2, 2.9 Hz, 1H), 2.86 – 2.61 (m, 2H), 2.00 – 1.86 (m, 1H), 1.56 (tdd, *J* = 12.7, 10.0, 5.6 Hz, 1H), 1.21 (d, *J* = 6.3 Hz, 3H). ^{13}C NMR (75 MHz, $CDCl_3$) δ [163.88, 160.69 (d, $^{1}J_{C\text{-}F}$ = 240.5 Hz)], [146.07, 145.93 (d, $^{3}J_{C\text{-}F}$ = 10.6 Hz)], [130.22, 130.09 (d, $^{3}J_{C\text{-}F}$ = 9.9 Hz)], [116.61, 116.58 (d, $^{4}J_{C\text{-}F}$ = 2.5 Hz)], [103.51, 103.23 (d, $^{2}J_{C\text{-}F}$ = 21.6 Hz)], [100.40, 100.07 (d, $^{2}J_{C\text{-}F}$ = 24.4 Hz)], 47.12, 30.11, 26.08, 22.58. MS (EI): *m/z* (rel. int.) 165.

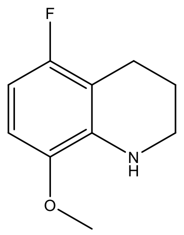

5-fluoro-8-methoxy-1,2,3,4-tetrahydroquinoline (2i): Yield 96% (The NMR spectra are consistent with the reported data).[1, 4, 15a] ^{1}H NMR (300 MHz, $CDCl_3$) δ 6.50 (dd, *J* = 8.8, 4.9 Hz, 1H), 6.26 (t, *J* = 9.0 Hz, 1H), 4.35 (br, 1H), 3.79 (s, 3H), 3.35 – 3.26 (m, 2H), 2.72 (t, *J* = 6.5 Hz, 2H), 1.94 (dtd, *J* = 8.9, 6.5, 4.8 Hz, 2H). ^{13}C NMR (75 MHz, $CDCl_3$) δ [157.70, 154.59 (d, $^{1}J_{C\text{-}F}$ = 234.3 Hz)], 142.36, [135.94, 135.83 (d, $^{3}J_{C\text{-}F}$ = 8.7 Hz)], [109.08, 108.77 (d, $^{2}J_{C\text{-}F}$ = 23.6 Hz)], [107.45, 107.32 (d, $^{3}J_{C\text{-}F}$ = 10.3 Hz)], [100.69, 100.37 (d, $^{2}J_{C\text{-}F}$ = 23.9 Hz)], 55.98, 41.01, 21.25, [20.00, 19.95 (d, $^{3}J_{C\text{-}F}$ = 3.8 Hz)]. MS (EI): *m/z* (rel. int.) 181.

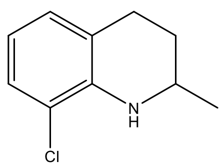

8-chloro-2-methyl-1,2,3,4-tetrahydroquinoline (2j): Yield 90% (The NMR spectra are consistent with the reported data).[1, 4, 15a, 18] ^{1}H NMR (300 MHz, $CDCl_3$) δ 7.08 (dt, *J* = 7.8, 1.1 Hz, 1H), 6.88 (dt, *J* = 7.5, 1.2 Hz, 1H), 6.53 (t, *J* = 7.7 Hz, 1H), 4.28 (br, 1H), 3.48 (dqd, *J* = 9.4, 6.3, 3.1 Hz, 1H), 2.95 – 2.69 (m, 2H), 1.96 (dddd, *J* = 12.5, 5.3, 3.9, 3.0 Hz, 1H), 1.60 (dddd, *J* = 12.9, 11.1, 9.8, 5.5 Hz, 1H), 1.29 (d, *J* = 6.2 Hz, 3H). ^{13}C NMR (75 MHz, $CDCl_3$) δ 140.86, 127.57, 126.85, 122.51, 117.95, 116.47, 47.29, 29.75, 26.88, 22.65. MS (EI): *m/z* (rel. int.) 181.

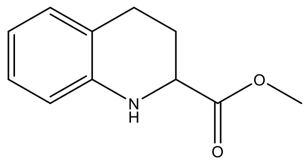

methyl 1,2,3,4-tetrahydroquinoline-2-carboxylate (2k): Yield 85% (The NMR spectra are consistent with the reported data).[4, 15a] ^{1}H NMR (300 MHz, $CDCl_3$) δ 7.06 – 6.92 (m, 2H), 6.63 (dd, *J* = 18.3, 11.4 Hz, 2H), 4.37 (br, 1H), 4.13 – 3.97 (m, 1H), 3.78 (s, 3H), 2.79 (tt, *J* = 16.6, 8.3 Hz, 2H), 2.37 – 2.23 (m, 1H), 2.02 (ddt, *J* = 13.3, 8.9, 4.5 Hz, 1H). ^{13}C NMR (75 MHz, $CDCl_3$) δ 173.82, 143.02, 129.20, 127.13, 120.67, 117.79, 114.71, 54.00, 52.42, 25.89, 24.81. MS (EI): *m/z* (rel. int.) 191.

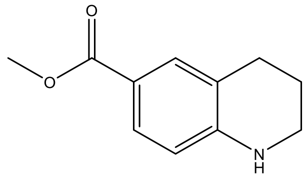

methyl 1,2,3,4-tetrahydroquinoline-6-carboxylate (2l): Yield 94% (The NMR spectra are consistent with the reported data).[4, 15a, 19] ^{1}H NMR (300 MHz, $CDCl_3$) δ 7.70 – 7.58 (m, 2H), 6.42 – 6.33 (m, 1H), 4.42 (br, 1H), 3.82 (d, *J* = 1.0 Hz, 3H), 3.36 – 3.27 (m, 2H), 2.74 (t, *J* = 6.4 Hz, 2H), 1.97 – 1.83 (m, 2H). ^{13}C NMR (75 MHz, $CDCl_3$) δ 167.61, 148.92, 131.29, 129.13, 119.87, 117.28, 112.65, 51.43, 41.71, 26.94, 21.41. MS (EI): *m/z* (rel. int.) 191.

5. REFERENCES

(1) Chen, F.; Surkus, A.-E.; He, L.; Pohl, M.-M.; Radnik, J.; Topf, C.; Junge, K.; Beller, M., Selective Catalytic Hydrogenation of Heteroarenes with N-Graphene-Modified Cobalt Nanoparticles (Co_3O_4–Co/NGr@α-Al_2O_3). *J. Am. Chem. Soc.* **2015,** *137* (36), 11718-11724.

(2) Wei, Z.; Chen, Y.; Wang, J.; Su, D.; Tang, M.; Mao, S.; Wang, Y., Cobalt Encapsulated in N-Doped Graphene Layers: An Efficient and Stable Catalyst for Hydrogenation of Quinoline Compounds. *Acs Catal.* **2016,** *6* (9), 5816-5822.

(3) Li, J.; Liu, G.; Long, X.; Gao, G.; Wu, J.; Li, F., Different active sites in a bifunctional Co@N-doped graphene shells based catalyst for the oxidative dehydrogenation and hydrogenation reactions. *J. Catal.* **2017,** *355*, 53-62.
(4) Sorribes, I.; Liu, L.; Doménech-Carbó, A.; Corma, A., Nanolayered Cobalt–Molybdenum Sulfides as Highly Chemo- and Regioselective Catalysts for the Hydrogenation of Quinoline Derivatives. *Acs Catal.* **2018,** *8* (5), 4545-4557.
(5) Sahoo, B.; Kreyenschulte, C.; Agostini, G.; Lund, H.; Bachmann, S.; Scalone, M.; Junge, K.; Beller, M., A robust iron catalyst for the selective hydrogenation of substituted (iso)quinolones. *Chem. Sci.* **2018,** *9* (42), 8134-8141.
(6) Wu, Y.; Chen, Z.; Cheong, W.-C.; Zhang, C.; Zheng, L.; Yan, W.; Yu, R.; Chen, C.; Li, Y., Nitrogen-coordinated cobalt nanocrystals for oxidative dehydrogenation and hydrogenation of N-heterocycles. *Chem. Sci.* **2019,** *10* (20), 5345-5352.
(7) Gong, W.; Yuan, Q.; Chen, C.; Lv, Y.; Lin, Y.; Liang, C.; Wang, G.; Zhang, H.; Zhao, H., Liberating N-CNTs Confined Highly Dispersed Co-N_x Sites for Selective Hydrogenation of Quinolines. *Adv. Mater.* **2019,** *31* (49), 1906051.
(8) Hervochon, J.; Dorcet, V.; Junge, K.; Beller, M.; Fischmeister, C., Convenient synthesis of cobalt nanoparticles for the hydrogenation of quinolines in water. *Catal. Sci. Technol.* **2020,** *10* (14), 4820-4826.
(9) Li, W.; Cui, X.; Junge, K.; Surkus, A.-E.; Kreyenschulte, C.; Bartling, S.; Beller, M., General and Chemoselective Copper Oxide Catalysts for Hydrogenation Reactions. *Acs Catal.* **2019,** *9* (5), 4302-4307.
(10) He, Z.-H.; Li, N.; Wang, K.; Wang, W.-T.; Liu, Z.-T., Selective hydrogenation of quinolines over a CoCu bimetallic catalyst at low temperature. *Mol. Catal.* **2019,** *470*, 120-126.
(11) Mikami, Y.; Ebata, K.; Mitsudome, T.; Mizugaki, T.; Jitsukawa, K.; Kaneda, K., Reversible Dehydrogenation-Hydrogenation of Tetrahydroquinoline-Quinoline Using a Supported Cooper Nanoparticle Catalyst. *Herocycles* **2011,** 82 (2), 1371-1377.
(12) Ryabchuk, P.; Agapova, A.; Kreyenschulte, C.; Lund, H.; Junge, H.; Junge, K.; Beller, M., Heterogeneous nickel-catalysed reversible, acceptorless dehydrogenation of N-heterocycles for hydrogen storage. *Chem. Commun.* **2019,** *55* (34), 4969-4972.
(13) Su, H.; Sun, L.-H.; Xue, Z.-H.; Gao, P.; Zhang, S.-N.; Zhai, G.-Y.; Zhang, Y.-M.; Lin, Y.-X.; Li, X.-H.; Chen, J.-S., Nitrogen-thermal modification of the bifunctional interfaces of transition metal/carbon dyads for the reversible hydrogenation and dehydrogenation of heteroarenes. *Chem. Commun.* **2019,** *55* (76), 11394-11397.
(14) Jaiswal, G.; Subaramanian, M.; Sahoo, M. K.; Balaraman, E., A Reusable Cobalt Catalyst for Reversible Acceptorless Dehydrogenation and Hydrogenation of N-Heterocycles. *ChemCatChem* **2019,** *11* (10), 2449-2457.
(15) (a) Adam, R.; Cabrero-Antonino, J. R.; Spannenberg, A.; Junge, K.; Jackstell, R.; Beller, M., A General and Highly Selective Cobalt-Catalyzed Hydrogenation of N-Heteroarenes under Mild Reaction Conditions. *Angew. Chem. Int. Ed.* **2017,** *56* (12), 3216-3220; (b) Wu, J.; Wang, C.; Tang, W.; Pettman, A.; Xiao, J., The Remarkable Effect of a Simple Ion: Iodide-Promoted Transfer Hydrogenation of Heteroaromatics. *Chem. Eur. J.* **2012,** *18* (31), 9525-9529.
(16) Chaudhaery, S. S.; Roy, K. K.; Shakya, N.; Saxena, G.; Sammi, S. R.; Nazir, A.; Nath, C.; Saxena, A. K., Novel Carbamates as Orally Active Acetylcholinesterase Inhibitors Found to Improve Scopolamine-Induced Cognition Impairment: Pharmacophore-Based Virtual Screening, Synthesis, and Pharmacology. *J. Med. Chem.* **2010,** *53* (17), 6490-6505.
(17) Ye, T.-N.; Li, J.; Kitano, M.; Hosono, H., Unique nanocages of 12CaO[middle dot]7Al_2O_3 boost heterolytic hydrogen activation and selective hydrogenation of heteroarenes over ruthenium catalyst. *Green Chem.* **2017,** *19* (3), 749-756.
(18) Talwar, D.; Li, H. Y.; Durham, E.; Xiao, J., A Simple Iridicycle Catalyst for Efficient Transfer Hydrogenation of N-Heterocycles in Water. *Chem. Eur. J.* **2015,** *21* (14), 5370-5379.
(19) Xuan, Q.; Song, Q., Diboron-Assisted Palladium-Catalyzed Transfer Hydrogenation of N-Heteroaromatics with Water as Hydrogen Donor and Solvent. *Org. Lett.* **2016,** *18* (17), 4250-4253.

6. ^{1}H NMR AND ^{13}C NMR SPECTRA OF THE ISOLATED PRODUCTS

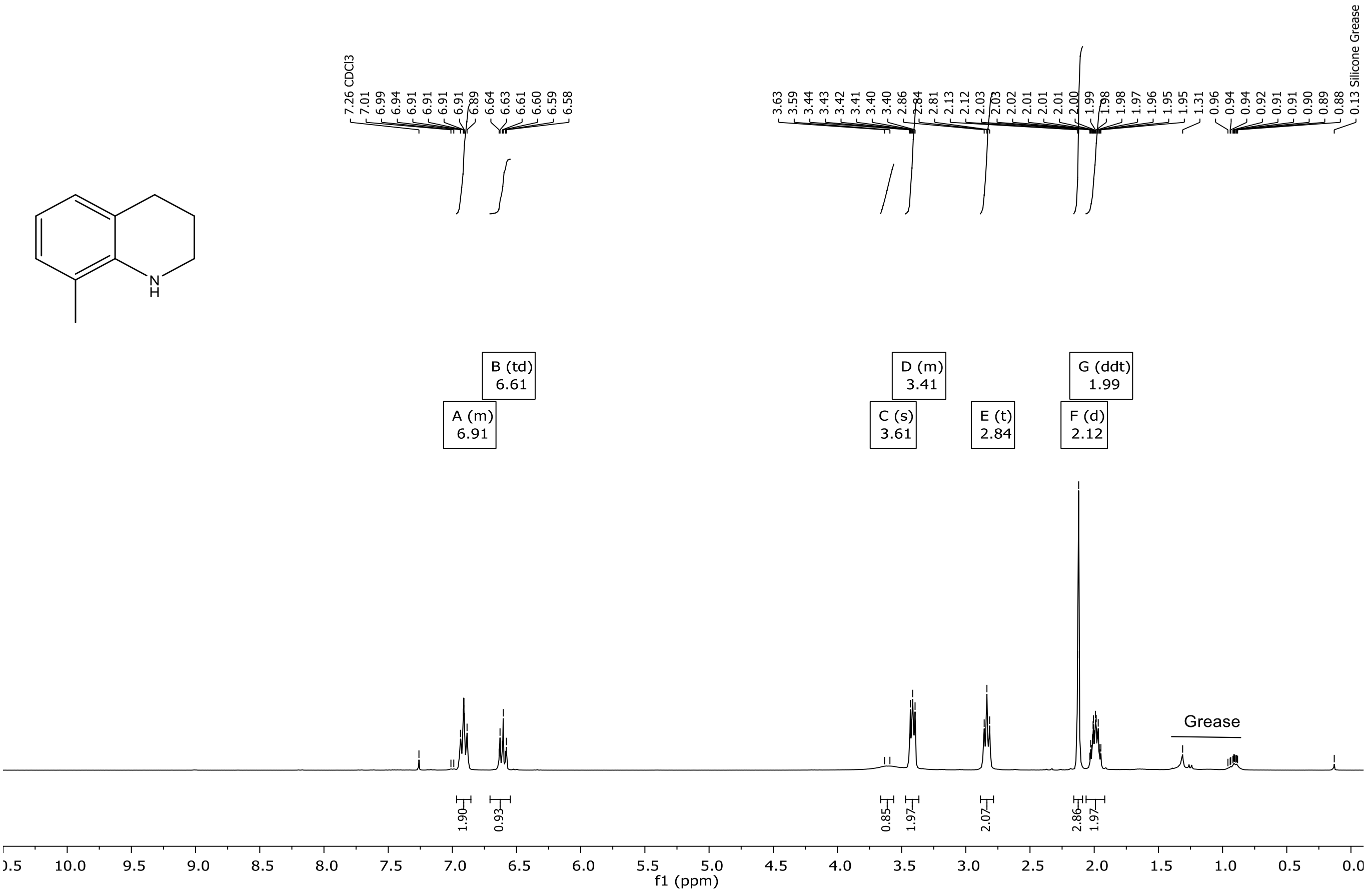

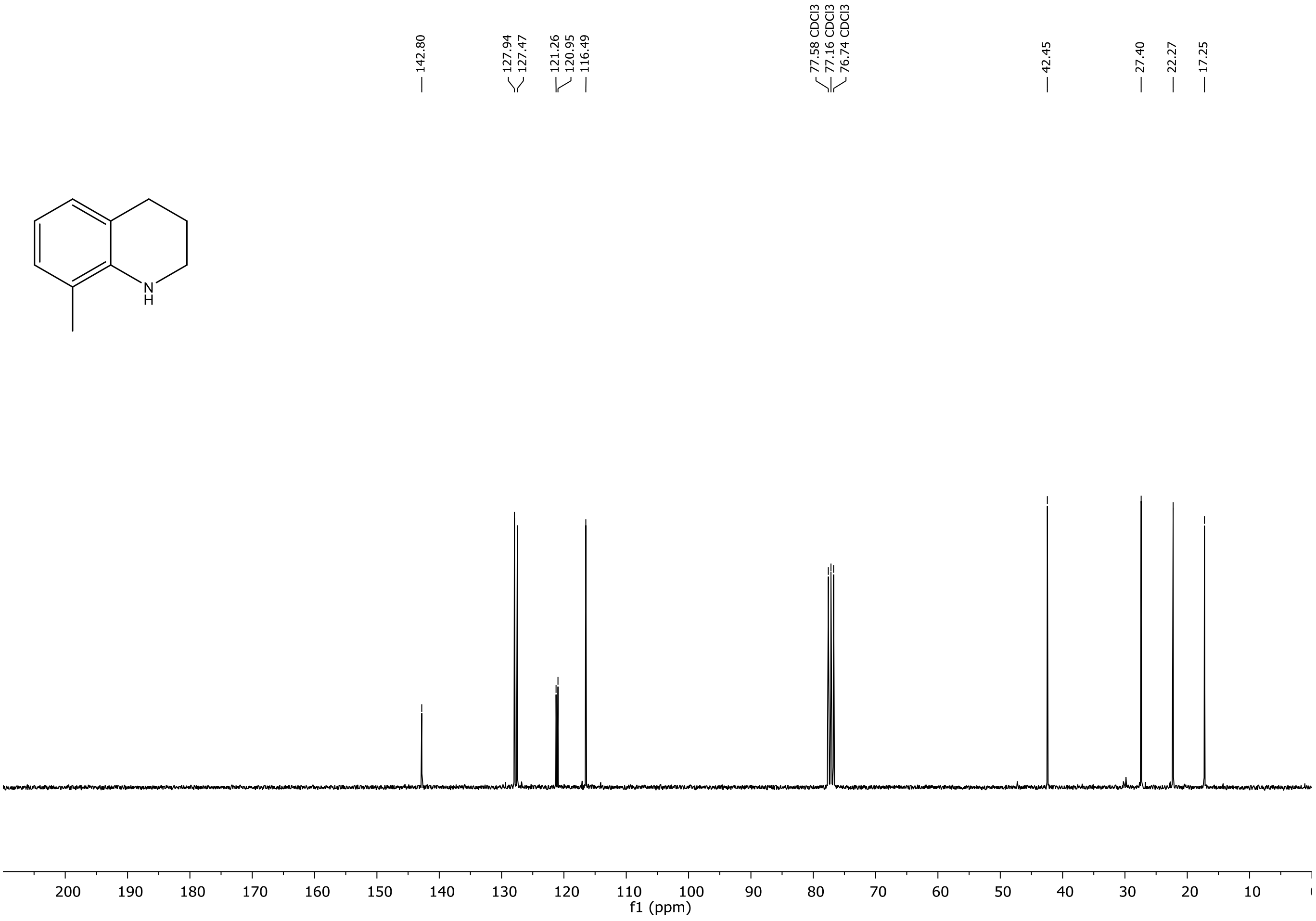

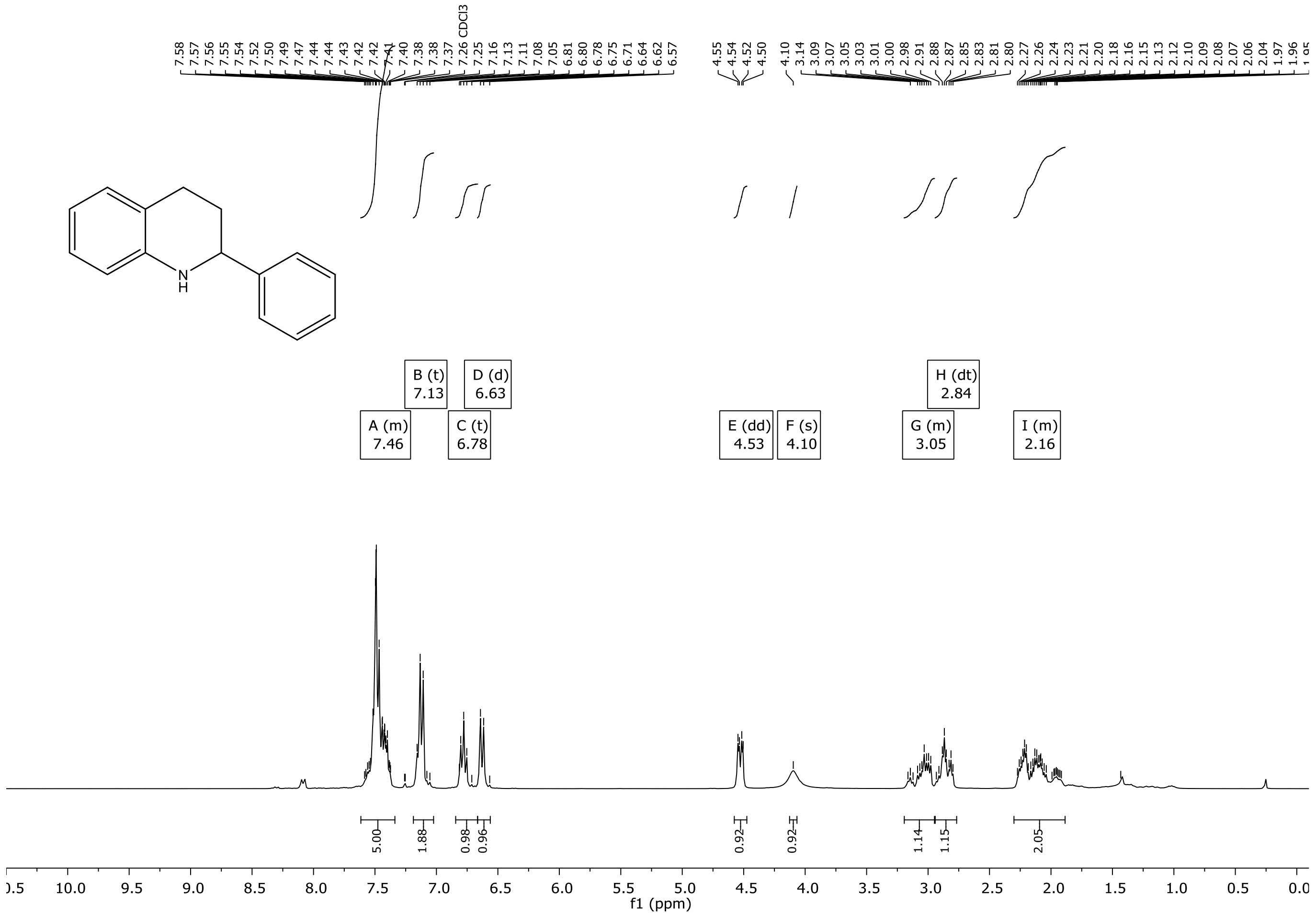
N
H
B (t) 7.13
D (d) 6.63
H (dt) 2.84
A (m) 7.46
C (t) 6.78
E (dd) 4.53
F (s) 4.10
G (m) 3.05
I (m) 2.16
5.00
1.88
0.98
0.96
0.92
0.92
1.14
1.15
2.05
f1 (ppm)

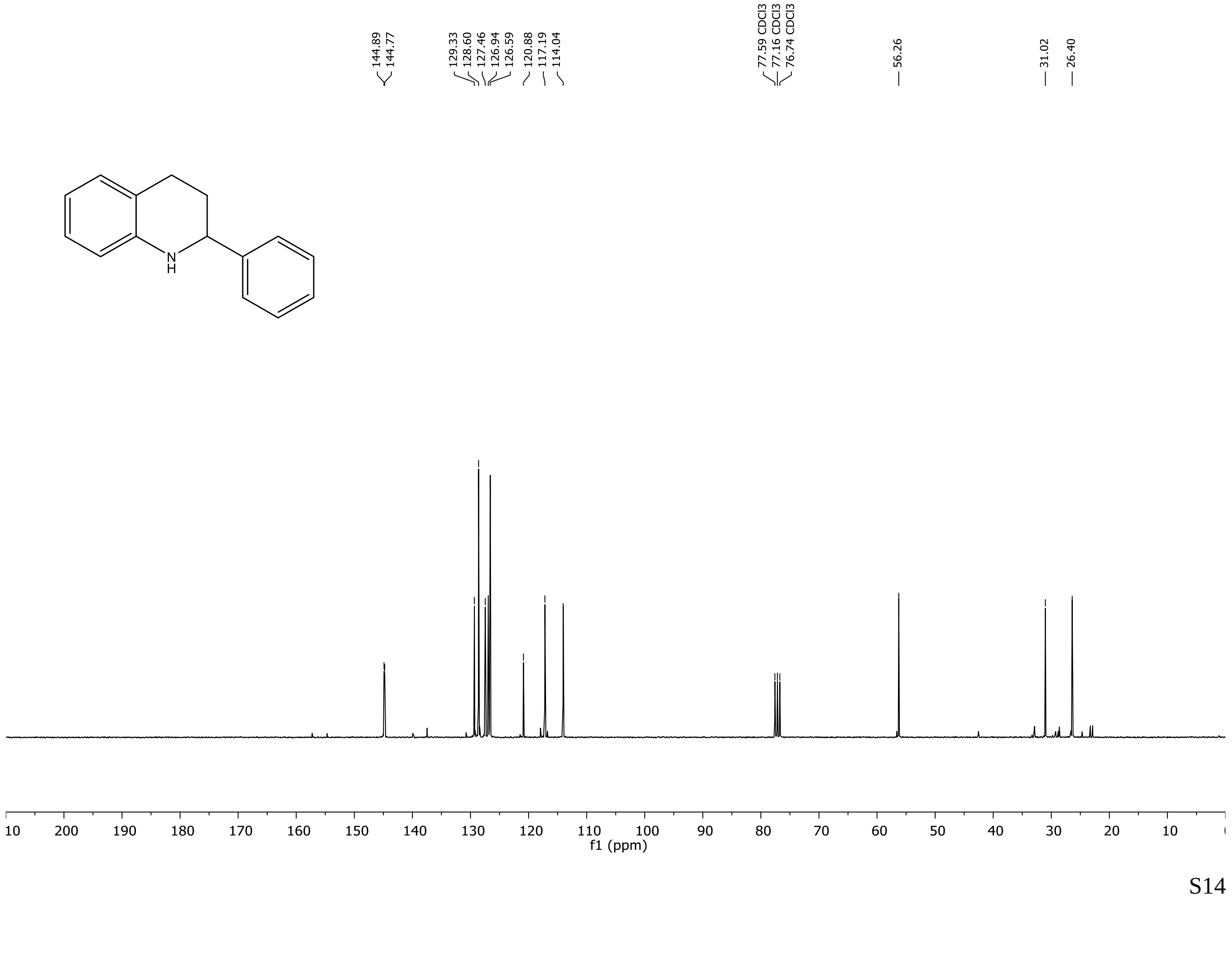
144.89
144.77
129.33
128.60
127.46
126.94
126.59
120.88
117.19
114.04
77.59 CDCl3
77.16 CDCl3
76.74 CDCl3
56.26
31.02
26.40
N
H
f1 (ppm)

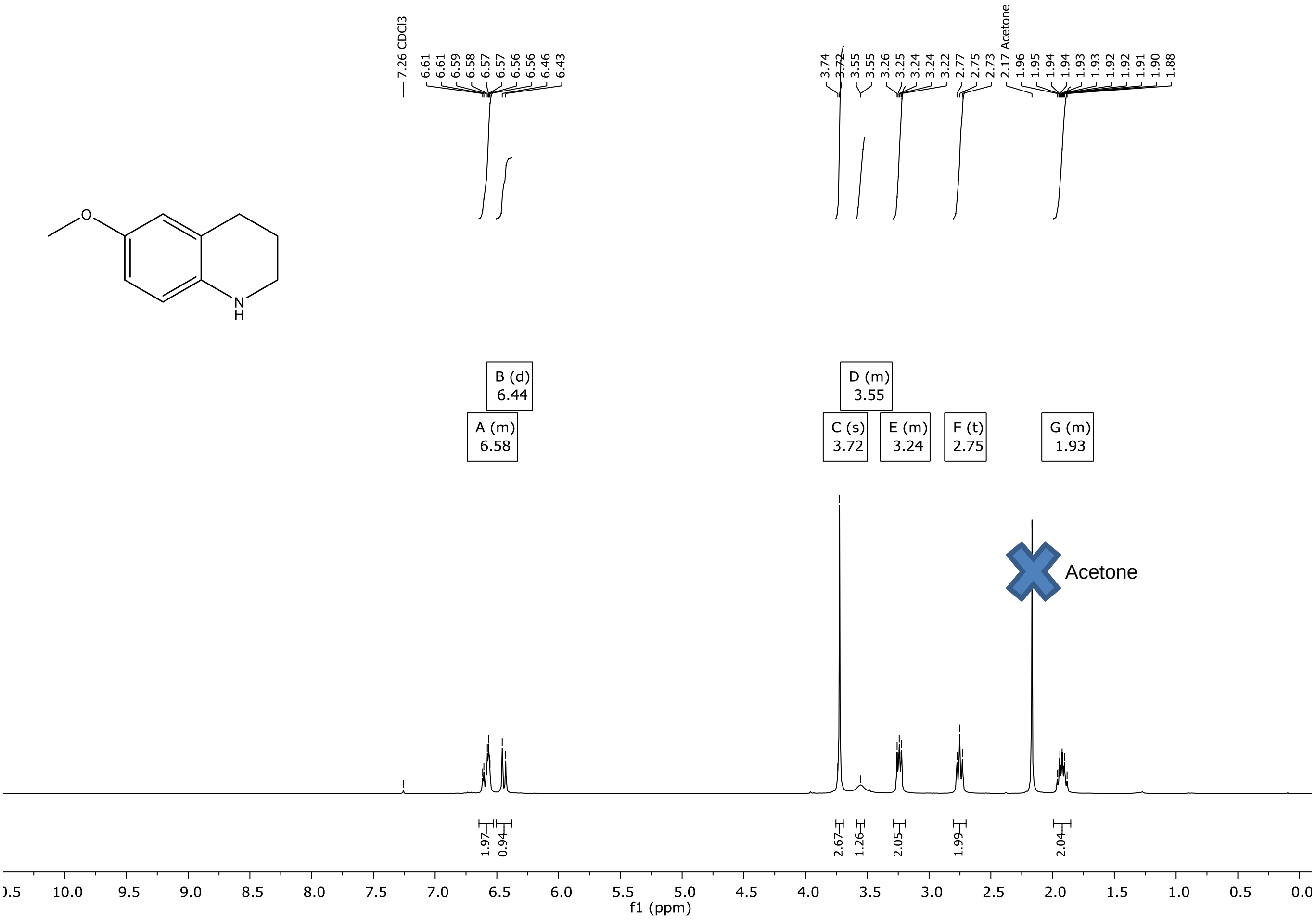
7.26 CDCl3
6.61
6.61
6.59
6.58
6.57
6.57
6.56
6.56
6.46
6.43
3.74
3.72
3.55
3.55
3.26
3.25
3.24
3.24
3.22
2.77
2.75
2.73
2.17 Acetone
1.96
1.95
1.94
1.94
1.93
1.93
1.92
1.92
1.91
1.90
1.88
O
N
H
B (d)
6.44
A (m)
6.58
D (m)
3.55
C (s)
3.72
E (m)
3.24
F (t)
2.75
G (m)
1.93
Acetone
1.97
0.94
2.67
1.26
2.05
1.99
2.04
f1 (ppm)

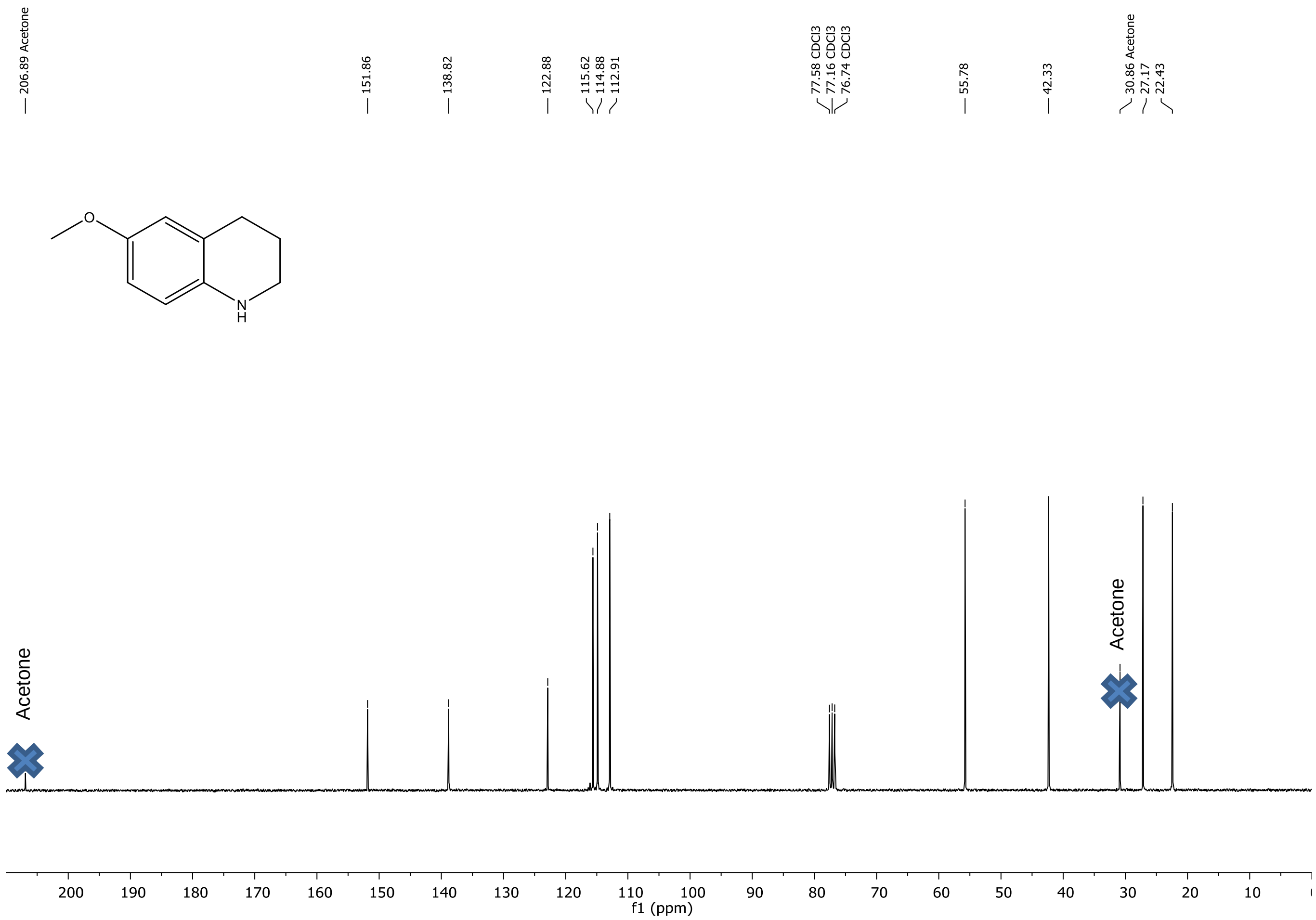
206.89 Acetone
151.86
138.82
122.88
115.62
114.88
112.91
77.58 CDCl3
77.16 CDCl3
76.74 CDCl3
55.78
42.33
30.86 Acetone
27.17
22.43
O
N
H
Acetone
Acetone
f1 (ppm)

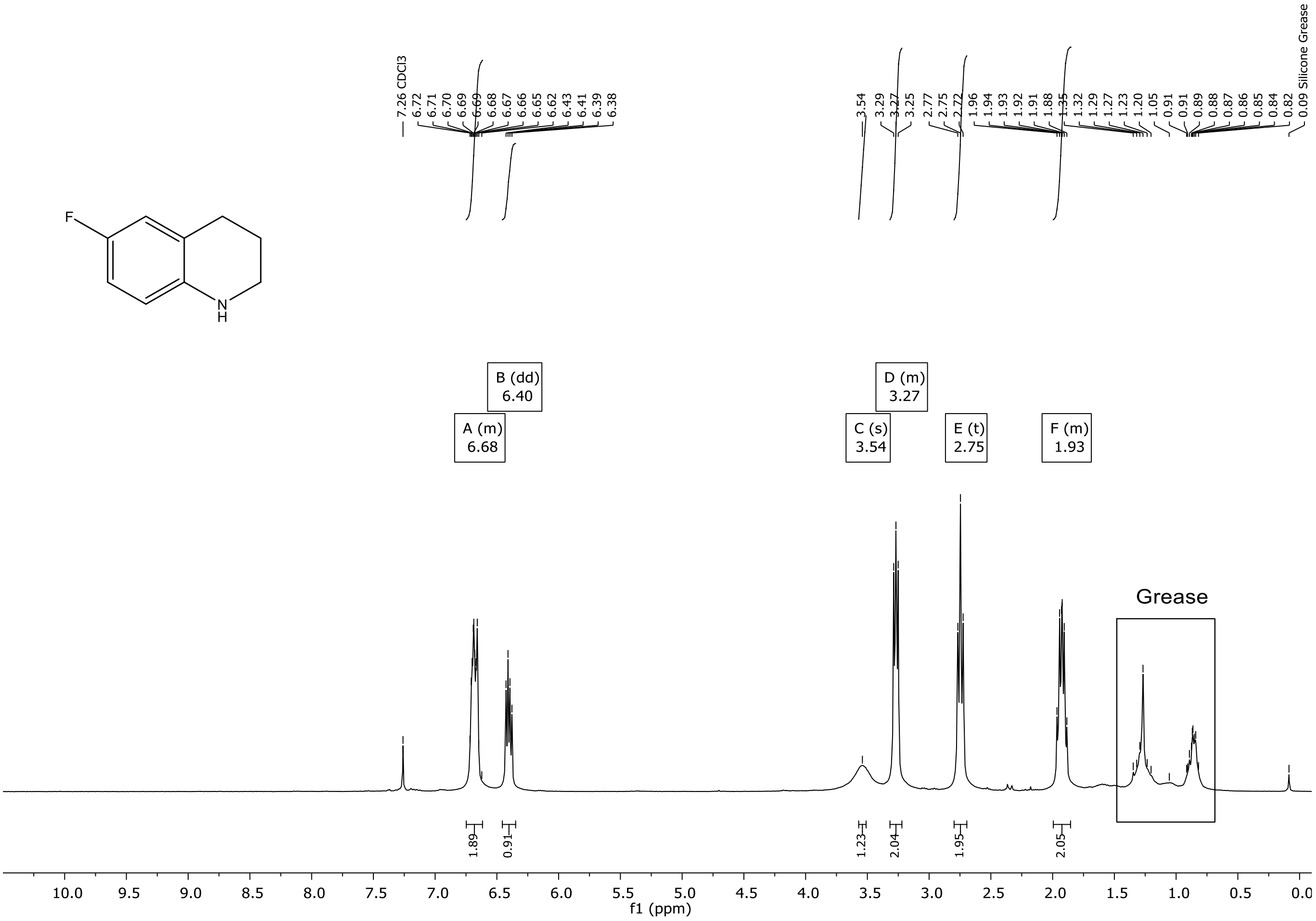
F
N
H
7.26 CDCl3
6.72
6.71
6.70
6.69
6.69
6.68
6.67
6.66
6.65
6.62
6.43
6.41
6.39
6.38
3.54
3.29
3.27
3.25
2.77
2.75
2.72
1.96
1.94
1.93
1.92
1.91
1.88
1.35
1.32
1.29
1.27
1.23
1.20
1.05
0.91
0.91
0.89
0.88
0.87
0.86
0.85
0.84
0.82
0.09 Silicone Grease
B (dd) 6.40
A (m) 6.68
D (m) 3.27
C (s) 3.54
E (t) 2.75
F (m) 1.93
Grease
1.89
0.91
1.23
2.04
1.95
2.05
f1 (ppm)

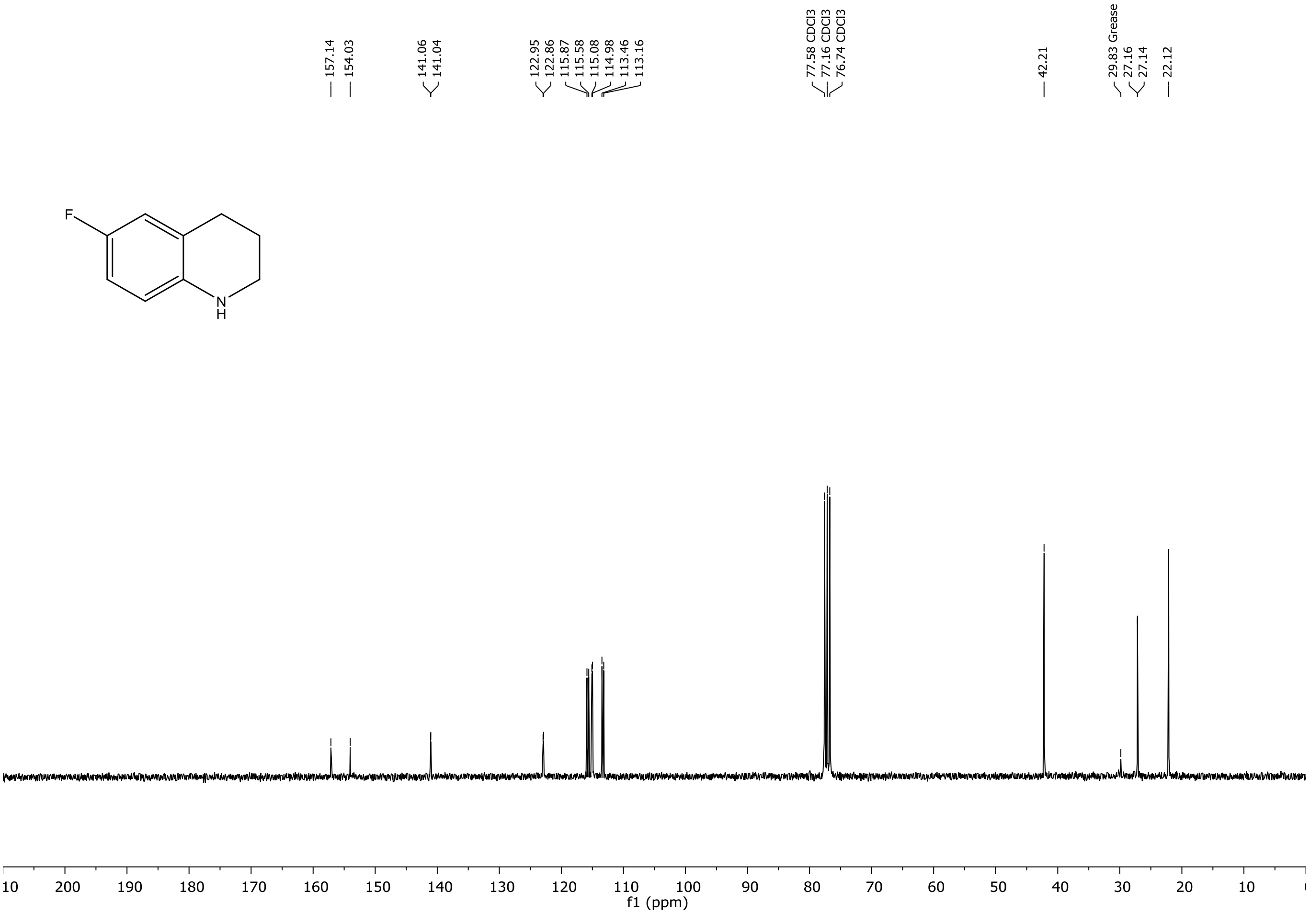
157.14
154.03
141.06
141.04
122.95
122.86
115.87
115.58
115.08
114.98
113.46
113.16
77.58 CDCl3
77.16 CDCl3
76.74 CDCl3
42.21
29.83 Grease
27.16
27.14
22.12
F
N
H
f1 (ppm)

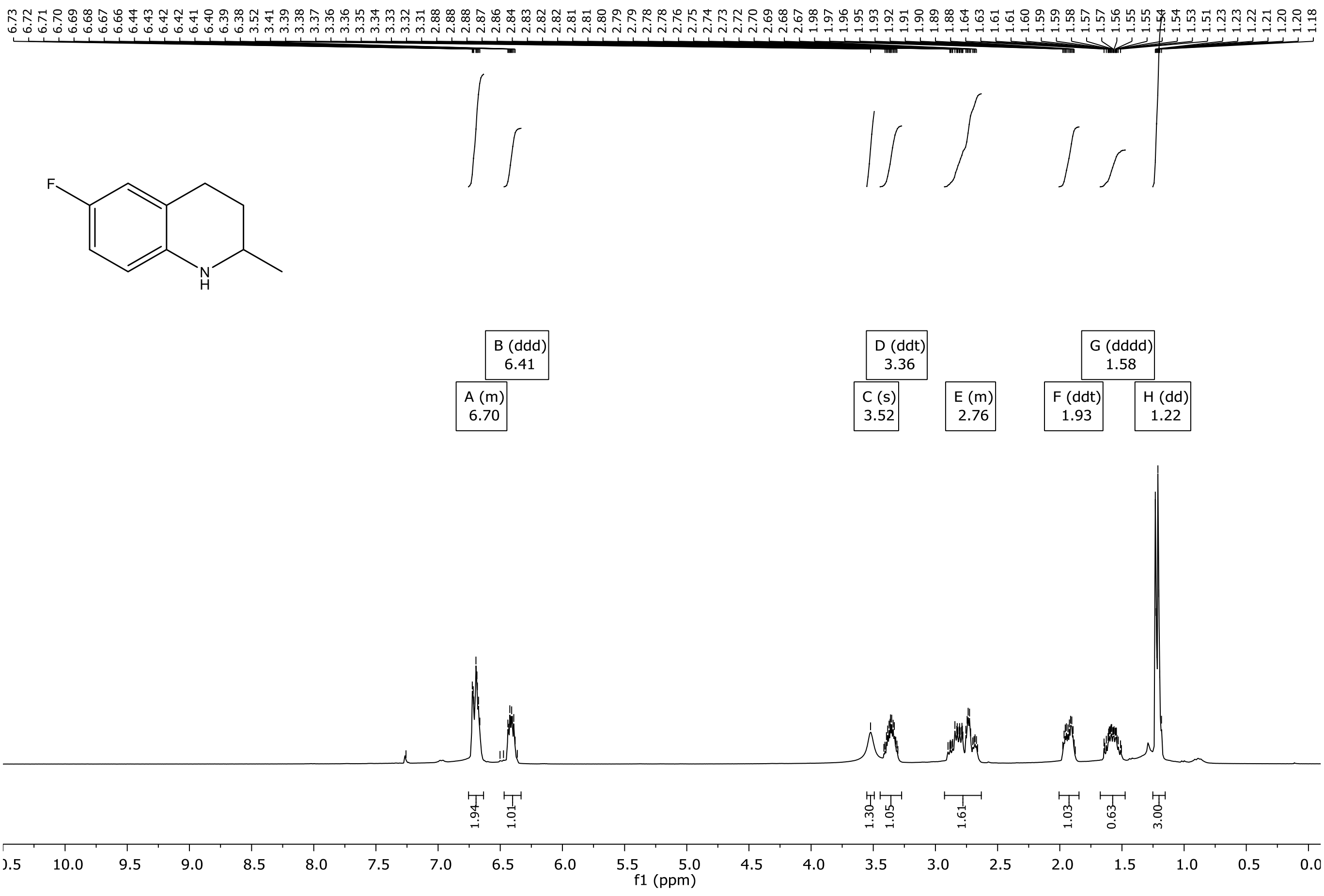
6.73 6.72 6.71 6.70 6.69 6.68 6.67 6.66 6.44 6.43 6.42 6.42 6.41 6.40 6.39 6.38 3.52 3.41 3.39 3.38 3.37 3.36 3.36 3.35 3.34 3.33 3.32 3.31 2.88 2.88 2.87 2.86 2.84 2.83 2.82 2.82 2.81 2.81 2.80 2.79 2.79 2.78 2.78 2.76 2.75 2.74 2.73 2.72 2.70 2.69 2.68 2.67 1.98 1.97 1.96 1.95 1.93 1.92 1.91 1.90 1.89 1.88 1.64 1.63 1.61 1.61 1.60 1.59 1.59 1.58 1.57 1.57 1.56 1.55 1.55 1.54 1.54 1.53 1.51 1.23 1.23 1.22 1.21 1.20 1.18
F
N
H
B (ddd) 6.41
A (m) 6.70
D (ddt) 3.36
C (s) 3.52
E (m) 2.76
G (dddd) 1.58
F (ddt) 1.93
H (dd) 1.22
1.94
1.01
1.30
1.05
1.61
1.03
0.63
3.00
f1 (ppm)

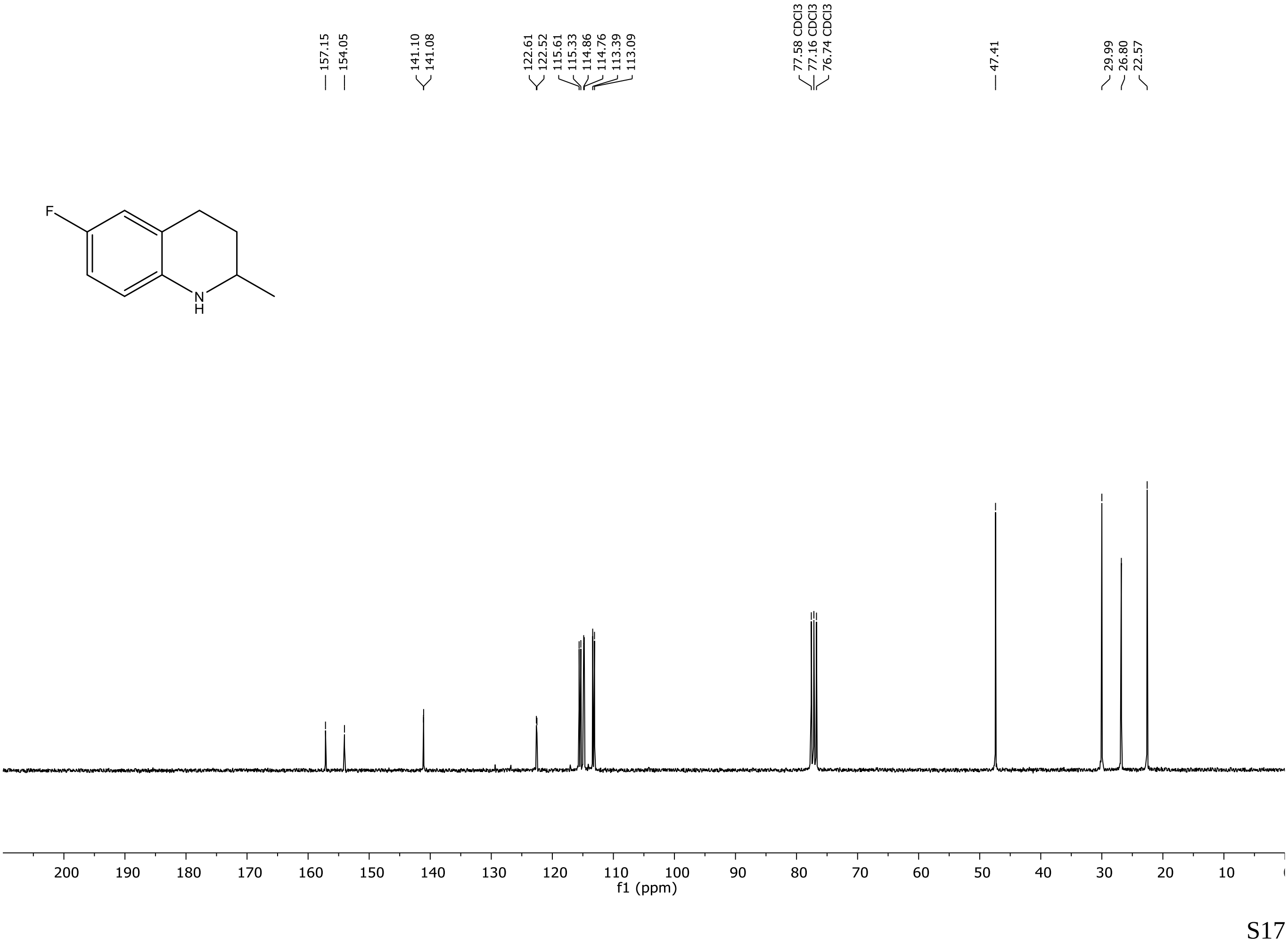
157.15
154.05
141.10
141.08
122.61
122.52
115.61
115.33
114.86
114.76
113.39
113.09
77.58 CDCl3
77.16 CDCl3
76.74 CDCl3
47.41
29.99
26.80
22.57
F
N
H
f1 (ppm)

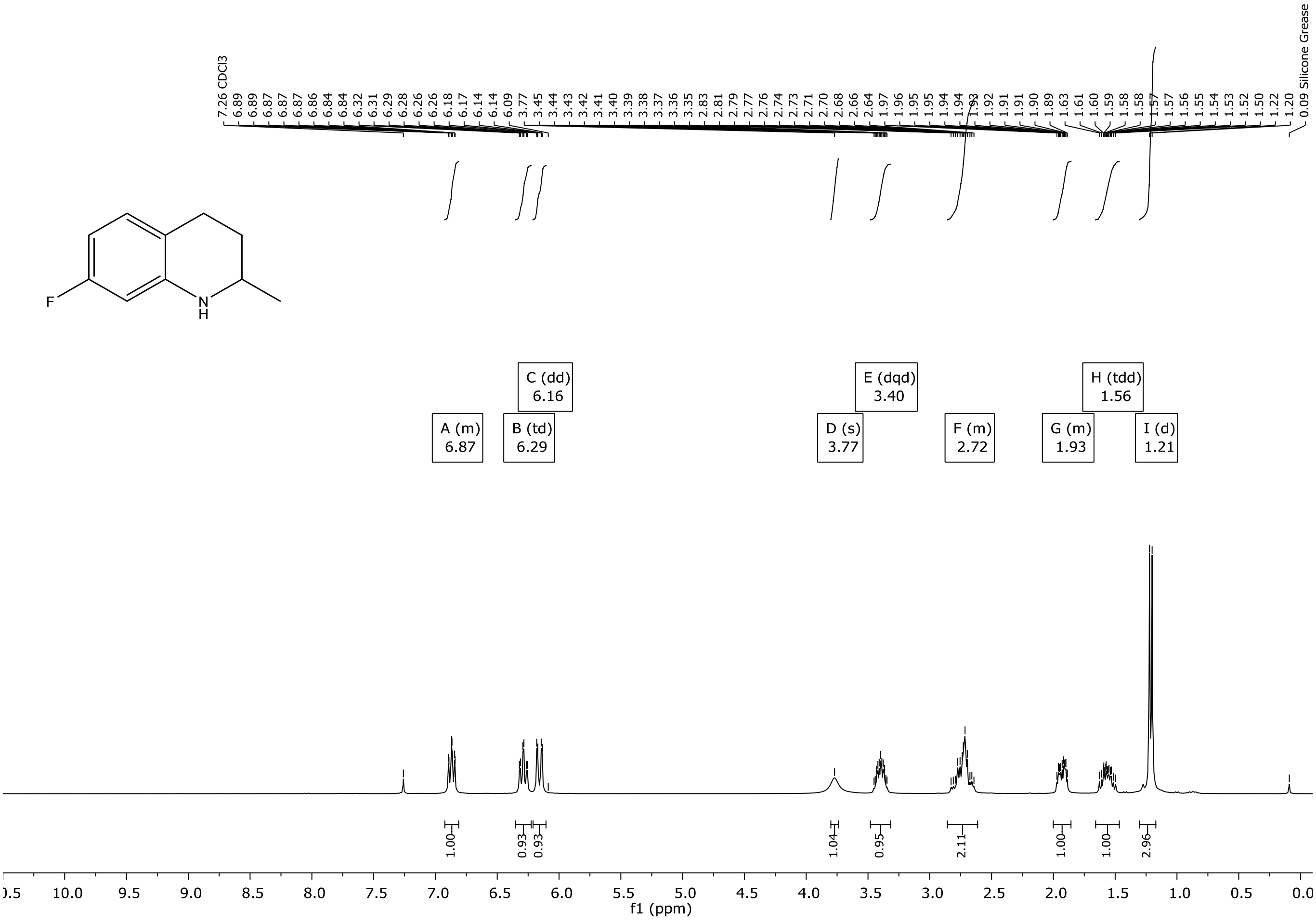

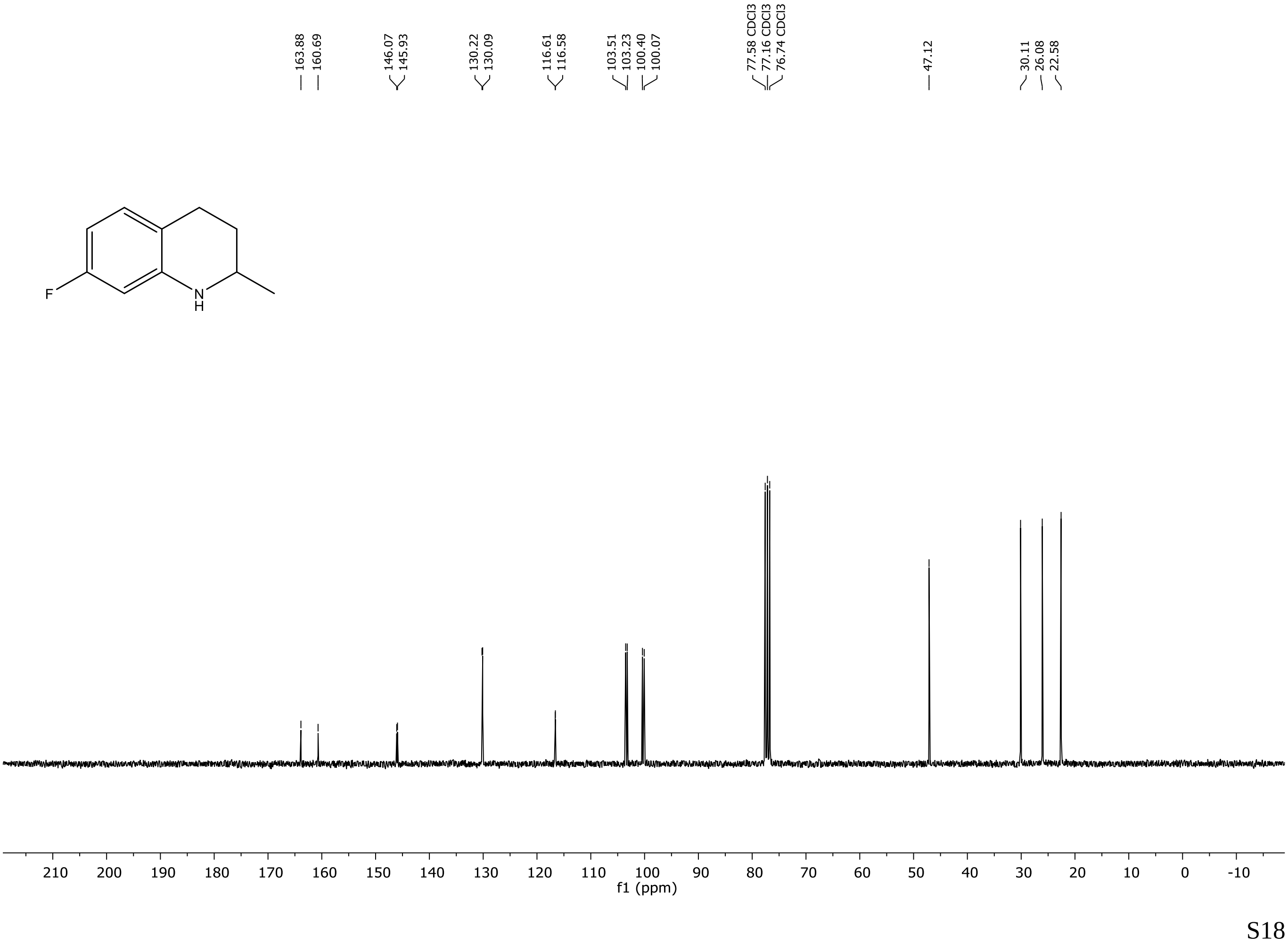

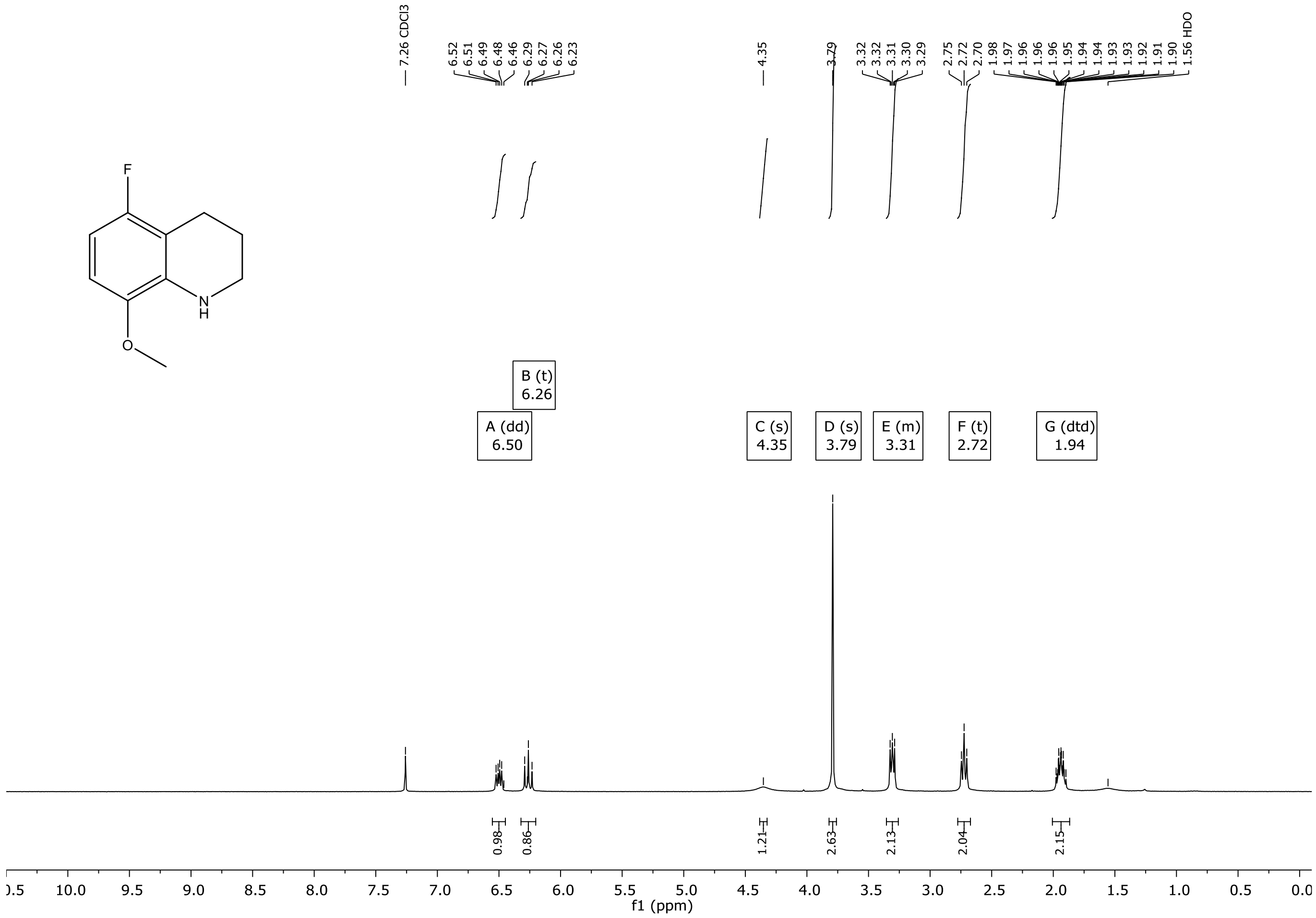
F
N
H
O
7.26 CDCl3
6.52 6.51 6.49 6.48 6.46 6.29 6.27 6.26 6.23
4.35
3.79
3.32 3.32 3.31 3.30 3.29
2.75 2.72 2.70
1.98 1.97 1.96 1.96 1.95 1.94 1.94 1.93 1.93 1.92 1.91 1.90
1.56 HDO
B (t) 6.26
A (dd) 6.50
C (s) 4.35
D (s) 3.79
E (m) 3.31
F (t) 2.72
G (dtd) 1.94
0.98 0.86 1.21 2.63 2.13 2.04 2.15
f1 (ppm)

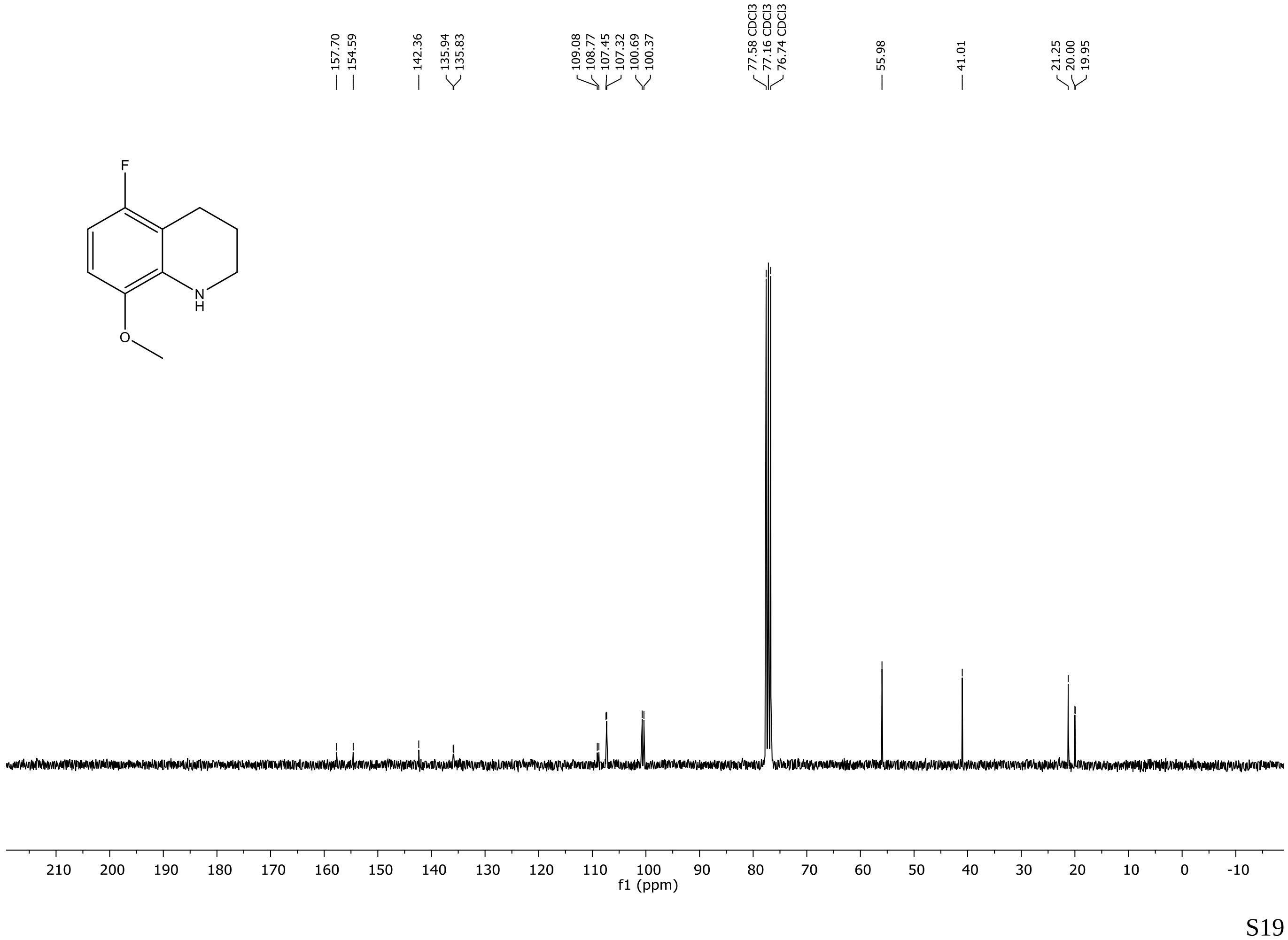
F
N
H
O
157.70 154.59
142.36 135.94 135.83
109.08 108.77 107.45 107.32 100.69 100.37
77.58 CDCl3 77.16 CDCl3 76.74 CDCl3
55.98
41.01
21.25 20.00 19.95
f1 (ppm)

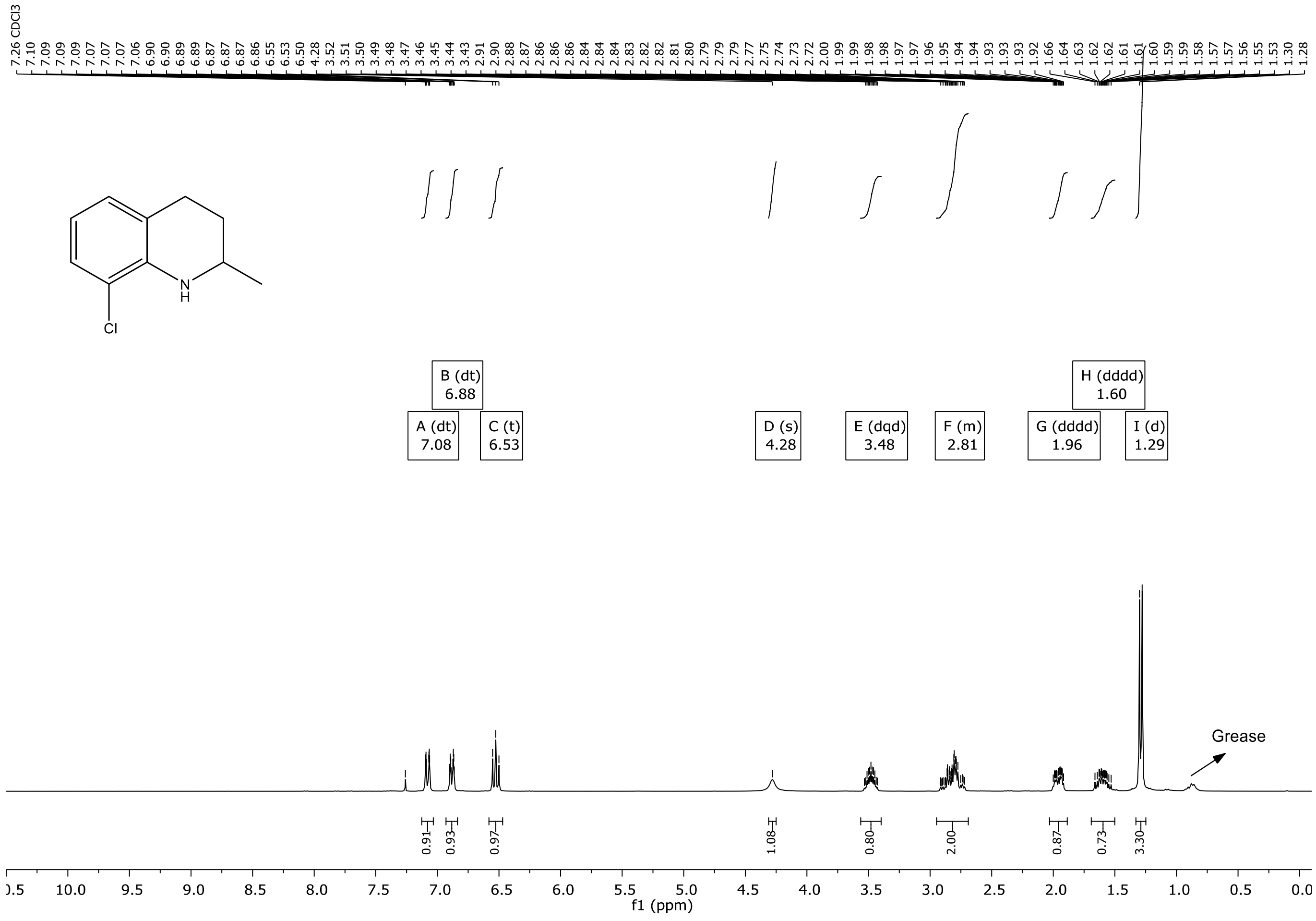
7.26 CDCl3 7.10 7.09 7.09 7.07 7.07 7.07 7.06 6.90 6.90 6.89 6.89 6.87 6.87 6.86 6.55 6.53 6.50 4.28 3.52 3.51 3.50 3.49 3.48 3.47 3.46 3.45 3.44 3.43 2.91 2.90 2.88 2.87 2.86 2.86 2.84 2.84 2.84 2.83 2.82 2.82 2.81 2.80 2.79 2.79 2.77 2.75 2.74 2.73 2.72 2.00 1.99 1.99 1.98 1.98 1.97 1.97 1.96 1.95 1.94 1.94 1.93 1.93 1.93 1.92 1.66 1.64 1.63 1.62 1.62 1.61 1.61 1.60 1.59 1.59 1.58 1.57 1.57 1.56 1.55 1.53 1.30 1.28
N
H
Cl
B (dt) 6.88
H (dddd) 1.60
A (dt) 7.08
C (t) 6.53
D (s) 4.28
E (dqd) 3.48
F (m) 2.81
G (dddd) 1.96
I (d) 1.29
Grease
0.91 0.93 0.97 1.08 0.80 2.00 0.87 0.73 3.30
f1 (ppm)

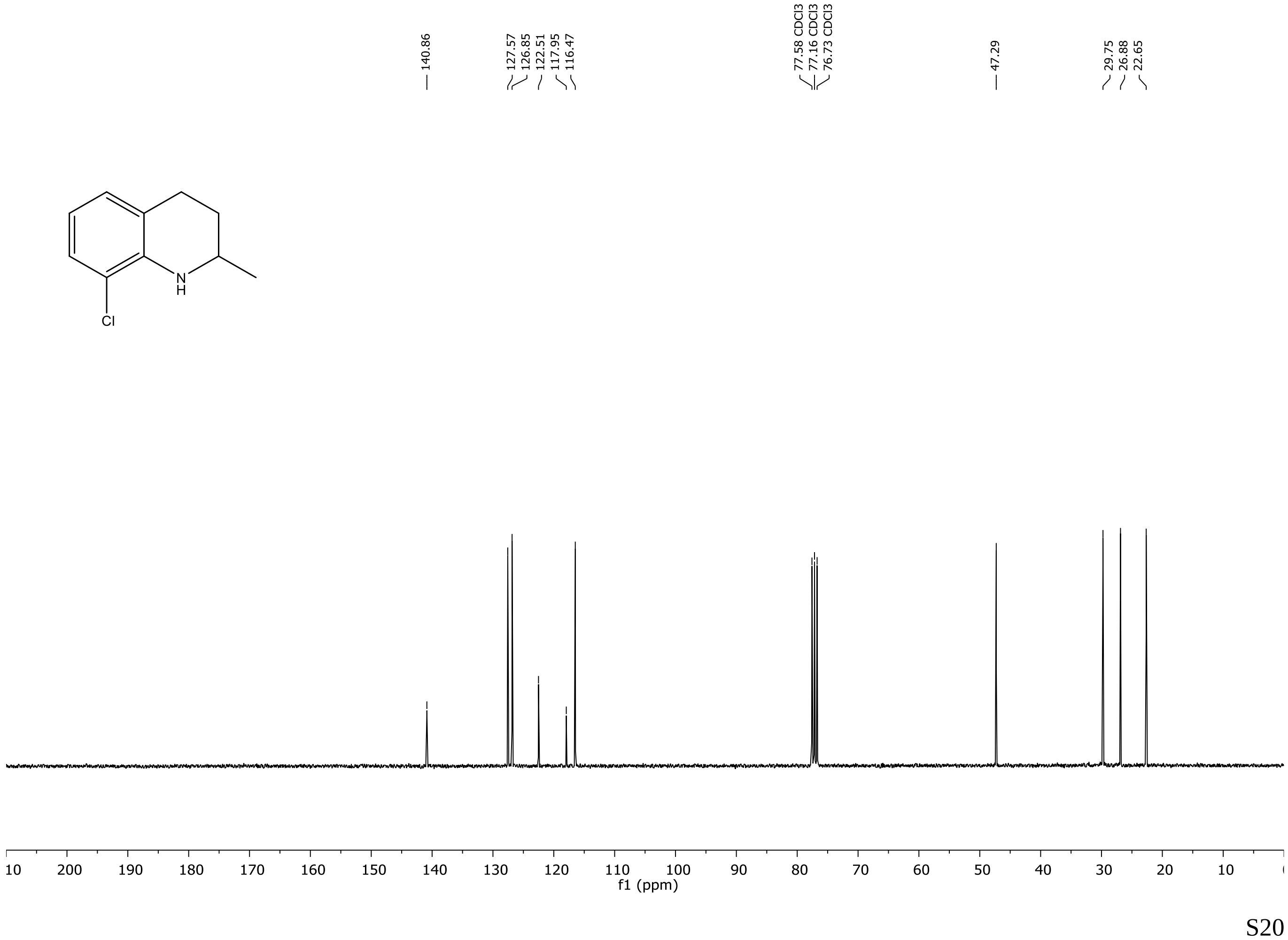
140.86
127.57 126.85 122.51 117.95 116.47
77.58 CDCl3 77.16 CDCl3 76.73 CDCl3
47.29
29.75 26.88 22.65
N
H
Cl
f1 (ppm)

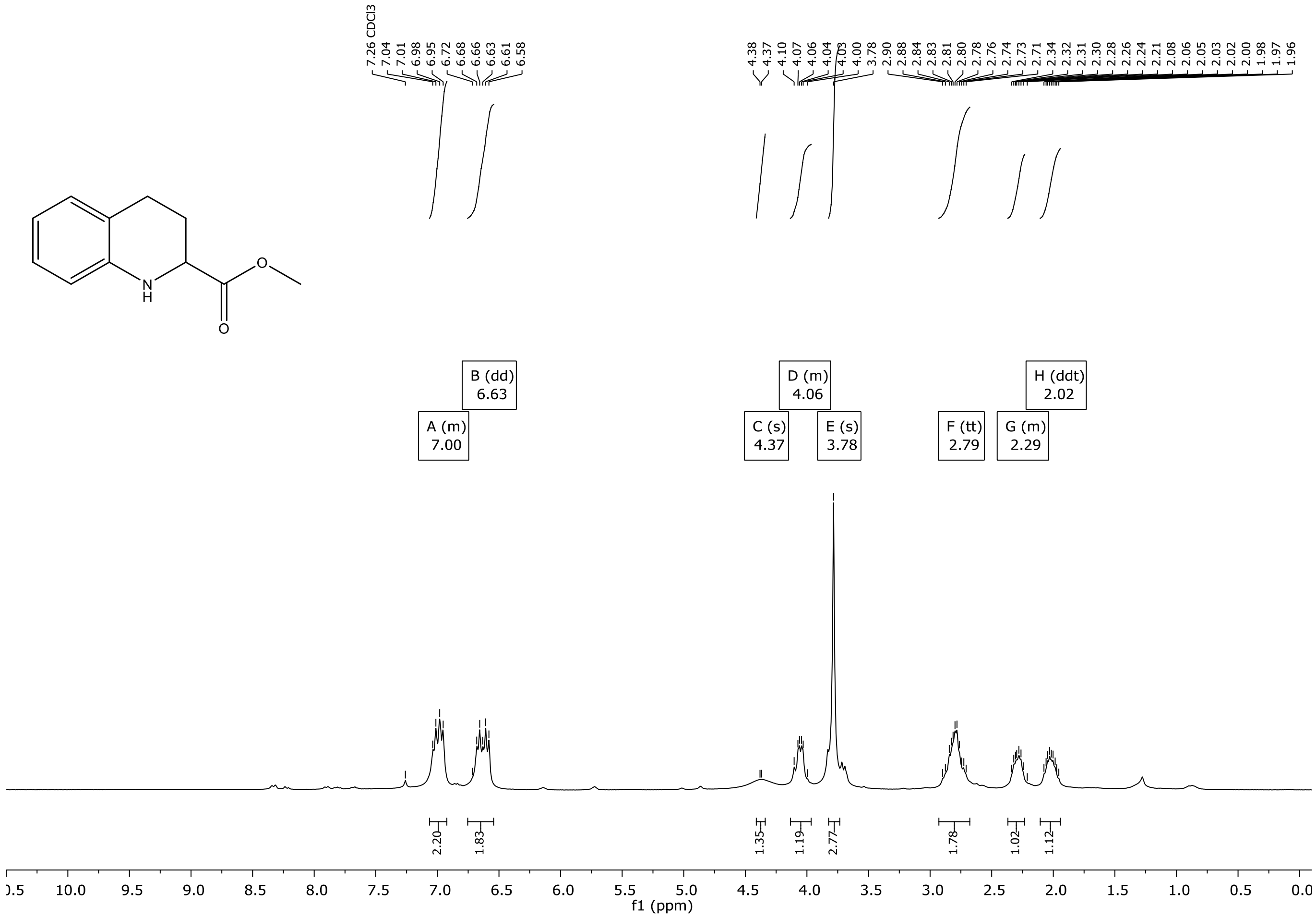
7.26 CDCl3
7.04
7.01
6.98
6.95
6.72
6.68
6.66
6.63
6.61
6.58
4.38
4.37
4.10
4.07
4.06
4.04
4.03
4.00
3.78
2.90
2.88
2.84
2.83
2.81
2.80
2.78
2.76
2.74
2.73
2.71
2.34
2.32
2.31
2.30
2.28
2.26
2.24
2.21
2.08
2.06
2.05
2.03
2.02
2.00
1.98
1.97
1.96
N
H
O
O
B (dd) 6.63
A (m) 7.00
D (m) 4.06
C (s) 4.37
E (s) 3.78
F (tt) 2.79
G (m) 2.29
H (ddt) 2.02
2.20
1.83
1.35
1.19
2.77
1.78
1.02
1.12
f1 (ppm)

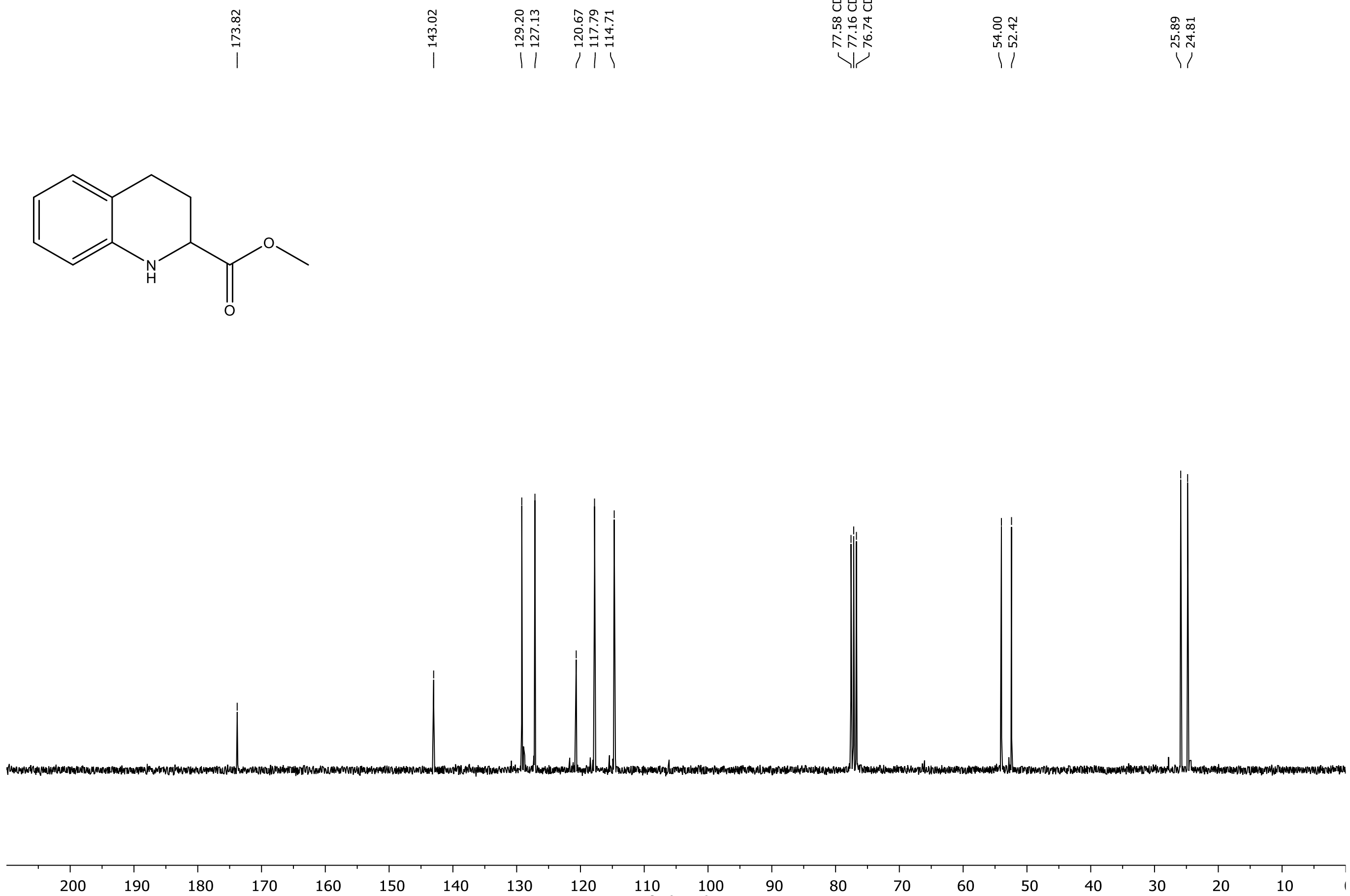
173.82
143.02
129.20
127.13
120.67
117.79
114.71
77.58 CDCl3
77.16 CDCl3
76.74 CDCl3
54.00
52.42
25.89
24.81
N
H
O
O
f1 (ppm)

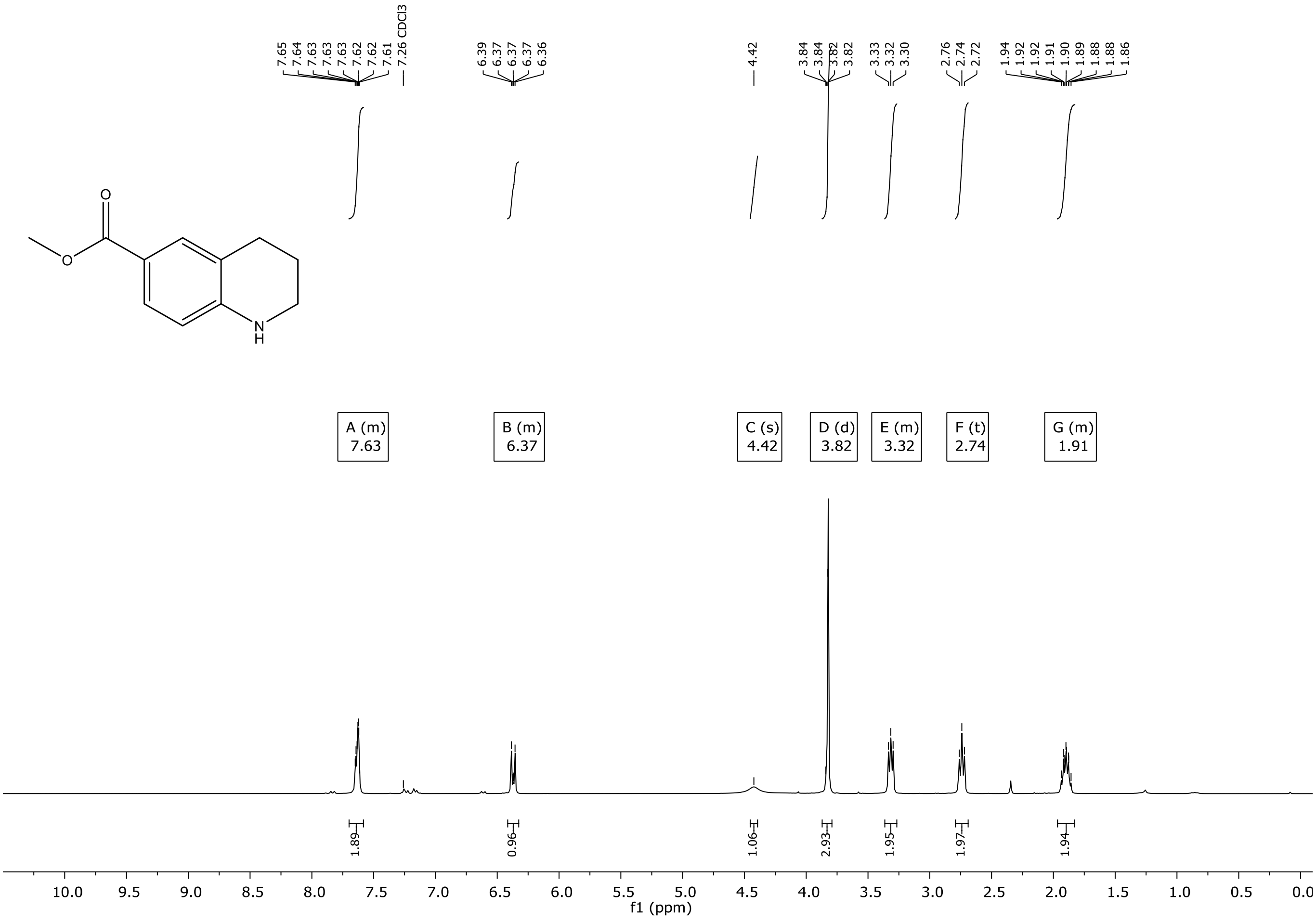
7.65
7.64
7.63
7.63
7.63
7.62
7.62
7.61
7.26 CDCl3
6.39
6.37
6.37
6.37
6.36
4.42
3.84
3.84
3.82
3.82
3.33
3.32
3.30
2.76
2.74
2.72
1.94
1.92
1.92
1.91
1.90
1.89
1.88
1.88
1.86
O
O
N
H
A (m) 7.63
B (m) 6.37
C (s) 4.42
D (d) 3.82
E (m) 3.32
F (t) 2.74
G (m) 1.91
1.89
0.96
1.06
2.93
1.95
1.97
1.94
f1 (ppm)

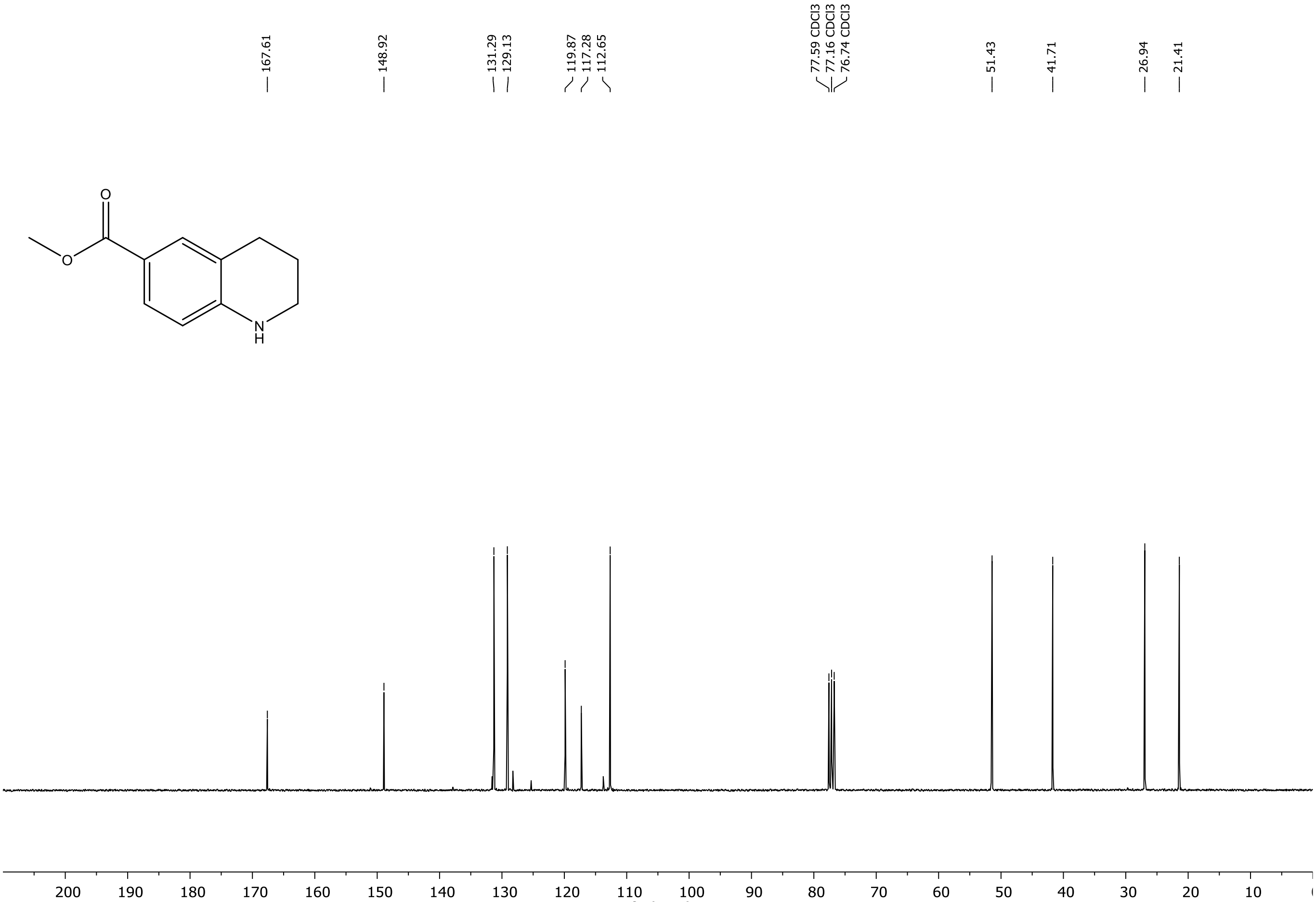
167.61
148.92
131.29
129.13
119.87
117.28
112.65
77.59 CDCl3
77.16 CDCl3
76.74 CDCl3
51.43
41.71
26.94
21.41
O
O
N
H
f1 (ppm)
